# Expression Patterns of *Anaplasma marginale msp2* Variants Change in Response to Growth in Cattle, and Tick Cells *versus* Mammalian Cells

**DOI:** 10.1371/journal.pone.0036012

**Published:** 2012-04-25

**Authors:** Adela S. Oliva Chávez, Roderick F. Felsheim, Timothy J. Kurtti, Pei-Shin Ku, Kelly A. Brayton, Ulrike G. Munderloh

**Affiliations:** 1 Department of Entomology, University of Minnesota, Saint Paul, Minnesota, United States of America; 2 Department of Veterinary Microbiology and Pathology, The Paul G. Allen School for Global Animal Health, Washington State University, Pullman, Washington, United States of America; Kansas State University, United States of America

## Abstract

Antigenic variation of major surface proteins is considered an immune-evasive maneuver used by pathogens as divergent as bacteria and protozoa. Likewise, major surface protein 2 (Msp2) of the tick-borne pathogen, *Anaplasma marginale*, is thought to be involved in antigenic variation to evade the mammalian host immune response. However, this dynamic process also works in the tick vector in the absence of immune selection pressure. We examined Msp2 variants expressed during infection of four tick and two mammalian cell-lines to determine if the presence of certain variants correlated with specific host cell types. *Anaplasma marginale* colonies differed in their development and appearance in each of the cell lines (P<0.001). Using Western blots probed with two Msp2-monospecific and one Msp2-monoclonal antibodies, we detected expression of variants with differences in molecular weight. Immunofluorescence-assay revealed that specific antibodies bound from 25 to 60% of colonies, depending on the host cell-line (P<0.001). Molecular analysis of cloned variant-encoding genes demonstrated expression of different predominant variants in tick (V1) and mammalian (V2) cell-lines. Analysis of the putative secondary structure of the variants revealed a change in structure when *A. marginale* was transferred from one cell-type to another, suggesting that the expression of particular Msp2 variants depended on the cell-type (tick or mammalian) in which *A. marginale* developed. Similarly, analysis of the putative secondary structure of over 200 Msp2 variants from ticks, blood samples, and other mammalian cells available in GenBank showed the predominance of a specific structure during infection of a host type (tick versus blood sample), demonstrating that selection of a possible structure also occurred *in vivo*. The selection of a specific structure in surface proteins may indicate that Msp2 fulfils an important role in infection and adaptation to diverse host systems. Supplemental Abstract in Spanish (File S1) is provided.

## Introduction

Several bacterial pathogens survive the complex mammalian adaptive immune system by changing their surface protein [1], [2]. This results in the establishment of persistent infection, enhancing their transmission to a susceptible host [3]. *Anaplasma marginale* is a tick-borne, obligate intracellular χ-proteobacterium in the order Rickettsiales, family Anaplasmataceae, that causes bovine anaplasmosis [4]. This pathogen utilizes a recombinatorial mechanism of antigenic variation in which different variants of the immunodominant major surface protein 2 (Msp2) are expressed during different phases of infection [2], [5], [6]. The course of disease is characterized by cyclical parasitemic peaks that follow the primary infection and persist during the life of the animal. These cycles in the infection are the result of the recognition and clearance of bacteria expressing a Msp2 variant by variant-specific host antibodies and the subsequent emergence of new variants [7]–. Both *A. marginale* and the closely related *A. phagocytophilum*, the causative agent of human granulocytic anaplasmosis, possess a polycistronic expression locus that encodes the single *msp2* expression cassette [11]. Dispersed throughout the chromosome, *A. marginale* encodes 7–12 *msp2* donor alleles (also referred as pseudogenes) with conserved regions flanking a central hypervariable region (HVR) [10]. In the early stages of disease, simple variants arise by duplication of an entire donor allele from the non-expressed site in the chromosome into the expression cassette. As infection continues, portions of multiple *msp2* donor alleles are recombined into the expression cassette by a gene conversion mechanism [2], [8], [10]. This last step results in a “mosaic" representing HVR sections of two or more donor alleles in the HVR of the expressed copy [10], [12].

Even though antigenic variation of this protein has primarily been associated with evasion of the immune response, *msp2* undergoes variation in the absence of immune selection within the tick vector [13]–[17]. Several authors have proposed that selection for new variants occurs in the tick after the blood meal and that some of these variants are unique to specific tissues, e.g. the salivary gland variants [15]–[17].

Variation in the *msp2* homolog *msp2(p44)* from *A. phagocytophilum*, has been shown to respond to changes in the environment in which the bacteria develop, i.e., in tick or mammalian cells [11]. Specific variants of *msp2(p44)* developed within 3 weeks of transferring the organism from mammalian cells to tick cells or vice versa. It has been suggested that *A. phagocytophilum* Msp2/P44 acts as a porin to facilitate acquisition of metabolites from the host cell [18]. It is possible that its homolog, Msp2, fulfils a similar role in *A. marginale*. Species- and tissue-specific differences in cell membrane structure could account for adaptive changes necessary to maintain function of the porin in the mammalian and tick tissues in which *Anaplasma* species replicate during completion of their life cycle. Antigenically variable proteins have been shown to be involved in tissue tropism in other bacteria, as in the case of VlsE in *Borrelia spp.* that is highly expressed during infection of mammalian cells (reviewed in [19]).

Palmer et al [6] proposed that selection for simple variants provided a fitness advantage to the organism when replicating in naïve animals and the tick vector. Generation of simple variants occurred within the first week of infection in naïve animals at a time when the immune system presumably had not yet encountered the complete repertoire of antigens encoded by genomic donor alleles. Donor alleles may undergo specific evolutionary selection for growth fitness [6] with certain variants preferentially expressed during early stages of acute infection. For example, 29% of the variants recovered during acute infection presented intact Msp2ψ1HVR or Msp2ψ1HVR containing a segmental change in its coding sequence [20].

We studied the variation of Msp2 during infection of different tick and mammalian cell lines with the *A. marginale* strain Virginia (*A. marginale* VA) using serologic and molecular approaches to determine if the host cell environment influenced expression of distinct variants. Herein, we report differences in development and morphology of *A. marginale* VA colonies in tick cell lines derived from different species that are competent natural or experimental vectors, and in two mammalian cell lines that support replication of *A. marginale* VA [21]. We discuss the possibility that these differences are linked to Msp2 variation as an adaptation to survival in distinct environments. We describe preferential expression of certain Msp2 variants during infection of specific host cells, similar to what has been observed with expression of Msp2/P44 in *A. phagocytophilum* [11], [13], [20], and selection for different conformational structures during infection of specific host cell types *in vitro* and *in vivo*. The preferential expression of certain Msp2 variants with specific structures in different host cells suggests that this protein may be involved in important interactions between *A. marginale* and its host cell.

## Materials and Methods

### A. marginale VA growth in cultured cells

For our studies on Msp2 expression, the *A. marginale* VA [22] was cultured in four tick cell lines at 34°C and two mammalian cell lines at 37°C in tightly capped flasks. The tick cell lines used were BME26 [23] derived from embryonated eggs of the southern cattle tick, *Rhipicephalus (Boophilus) microplus* (Canestrini), ISE6 [24] and IDE12 [25] derived from embryonated eggs of the blacklegged tick, *Ixodes scapularis* (Say), and DAE100T [26] from embryonated eggs of the Rocky Mountain wood tick, *Dermacentor andersoni* (Stiles). The mammalian cells used in this research were Vero (ATCC CCL-81 from kidney epithelial cells of an African Green monkey, *Cercopithecus aethiops*) and RF/6A cells (ATCC CRL-1780 from retina choroid endothelium of a rhesus monkey, *Macaca mulatta*). Uninfected tick cell cultures were inoculated with *A. marginale* VA [21] from ISE6 cells at passage 50, and mammalian cell lines were inoculated with cell free *A. marginale* VA purified from ISE6 cells at passage 46 [27]. Cell cultures were maintained in L15B300 medium as described previously [28].

### Bovine blood stabilates of A. marginale VA used for msp2 characterization

Frozen blood stabilate from a splenectomised calf, PA291, infected with *A. marginale* VA was regenerated from liquid nitrogen [21]. For comparison we used a second frozen blood stabilate taken from an infected 3 months old splenectomised Hereford calf, PA344, that had been inoculated with *A. marginale* VA grown in the *I. scapularis* cell line IDE8 [21]. A detailed description of the blood stabilates can be found in Materials and Methods S1. Frozen blood was rapidly thawed (37°C water bath), transferred to a 15 ml tube containing 10 ml L15B medium and gently mixed. The cell suspension was centrifuged at 2000×g for 10 min to remove DMSO and plasma. The cell pellet was resuspended in 10 ml L15B medium and the cells were washed twice more by centrifugation at 2000×g for 10 min. DNA was extracted from the cell fraction as described below (*Cloning and analysis of msp2 variants expressed in vitro and in vivo*).

### Anaplasma culture and evaluation of differences in development

After 5–6 passages of the bacteria in each of the cell lines, differences in the replication of *A. marginale* VA were assessed by measuring changes in the rate of cell-to-cell spread of the bacteria (infection rate). Infection rate was calculated as the slope of the linear regression line fitted to the percent infected cells at different time points from day 3 until the culture reached ≥90% infection (3, 5, 7, and 12 days post-inoculation, and at subculture). Cultures were monitored by light microscopic observation of Giemsa-stained cells [21] and the percent infected cells at each time point was determined by counting a total of 300 cells in four replicates (each replicate representing a separate infected culture) per cell line. Statistical differences in growth rates were evaluated using Repeated Measures ANOVA with SigmaPlot (Systat Software, Inc., San José, California).

### Western blots

Expression of Msp2 variants during infection of the different cell lines was analyzed by Western blotting. Protein was extracted from cell free bacteria purified from each cell line [27], and negative controls consisted of proteins extracted from uninfected IDE12 and ISE6 cells. Proteins were resolved by electrophoresis through polyacrylamide gels, and blotted onto membranes [27]. Blots were incubated with various Msp2 specific antibodies (Table 1) diluted in PBS with 3% bovine serum albumin overnight at 6°C, washed four times in PBS, and labelled with anti-rabbit or anti-mouse IgG conjugated to horseradish peroxidase, as required. Blots were developed with the ImmunoPure® metal enhanced DAB substrate (Pierce, Rockford, Illinois) system. Additional details about the methods used are explained in Materials and Methods S1.

**Table 1:** Antibodies used to serologically determine the variation of Msp2 in the different tick and mammalian cell lines.

Antibody	Type and specificity	Description	Dilution for western blot	Dilution for IFA	Reference
VMRD O50A2	Monoclonal anti-Msp2	Mouse IgG1. Capable of recognizing variants shared between different isolates	1∶200	1∶100	McGuire *et al.* (1984)
R883	Mono-specific anti-Msp2	Rabbit isotype not known. Animal inoculated with whole Msp2 from Florida isolate	1∶1000	1∶500	Palmer *et al.* (1988)
R884	Mono-specific anti-Msp2	Rabbit isotype not known. Animal inoculated with whole Msp2 from Florida isolate	1∶1000	1∶500	Palmer *et al.* (1988)

### Immuno-fluorescence assay (IFA)

Samples from triplicate cultures infected with *A. marginale* VA at 50% were spun onto microscope slides, and cell spots were then processed for IFA [28]. Uninfected cells and infected cells not exposed to primary antibody served as controls. The slides were dried, the cell spots were counter-stained with Evan's blue (0.0005%) and covered with VectaShield mounting medium with 4′-6-Diamidino-2-phenylindole (DAPI) (Vector laboratories, Burlingame, California). DAPI was used to stain DNA from all *A. marginale* VA and host cell nuclei. The percentage of colonies that bound a specific antibody was determined by counting 100 infected cells (counted using the host nuclei). Two-way ANOVA (SigmaPlot; Systat Software, Inc., San José, California) was carried out to evaluate the significance of the differences in antibody binding between *A. marginale* VA growing in the various cell lines. A detailed description of the procedures used for IFAs is given in Materials and Methods S1.

### Cloning and analysis of msp2 variants expressed in vitro and in vivo

After approximately 20 continuous passages in the same cell line, host cell-free *A. marginale* VA were harvested from cells grown in 25-cm^2^ flasks and bacterial DNA was extracted according to the tissue culture protocol of the Gentra Puregene genomic DNA kit [27] (Qiagen, Valencia, California). To confirm infection, *A. marginale* VA was PCR-amplified using primers listed in Table 2. Additionally, we extracted DNA from the previously mentioned blood samples. DNA from infected bovine blood (PA291 and PA344) was extracted following the whole blood protocol of the Gentra Puregene DNA kit. PA291 DNA was additionally treated with GeneReleaser, according to manufacturer's specifications (BioVentures Inc., Murfreesboro, Tennessee). More information about the blood samples is provided in Materials and Methods S1.

**Table 2:** Primers used for the confirmation of *A. marginale* infection, PCR amplification of Msp2 variants, and Msp2 expression qRT-PCR analysis.

Primer Specificity	Gene target	Product size	Nucleotide sequence	Ref
		(bp)		
*Anaplasma*	*16 s rRNA*	451	PER1 5′-TTT ATC GCT ATT AGA TGA GCC TAT G-3′	Goodman et al., 1996
*or Ehrlichia*			PER2 5′-CTC TAC ACT AGG AAT TCC GCT AT-3′	
*A. marginale*	*msp1β*	407	BAP-2 5′-GTA TGG CAC GTA GTC TTG GGA TCA-3′	Stich et al., 1993
			AL34S 5′-CAG CAG CAG CAA GAC CTT CA-3′	
*A. marginale*	*msp2*	1,125	For 5′-TCC TAC CAA GAG TCT TTT CCC C-3′	Futse et al., 2005
	*expression*		Rev 5′-TTA CCA CCG ATA CCA GCA CAA-3′	
*A. marginale*	*msp2 HVR*	∼366	qRT-PCRf 5′-AGT ATT GGA GGA GCC AGG GT-3′	Herein
			qRT-PCRr 5′-GTC CAT TGA CGA TAT GGC CT-3′	
*A. marginale*	*RNA polymerase*	282	Forward 5′-CGA ACT CAG GAA ACT GCT CC-3′	Herein
			Reverse 5′- AAA TTG TGC TTA ACC GCC AC-3′	
*A. marginale*	*16 s rRNA*	193	Forward 5′-TCT TAA CAG AAG GGC GCA GT-3′	Herein
			Reverse 5′- GAC TTG ACA TCA TCC CCA CC-3′	
*A. marginale*	*23 s rRNA*	172	Forward 5′-CCG GTG CTG GAA GGT TAA TA-3′	Herein
			Reverse 5′-AAT TTC GCT GAG TCG ATG CT-3′	

The *msp2* gene copy in the expression cassette was amplified using PCR with primers listed in Table 2 and under conditions described in Materials and Methods S1 [29]. Amplified products were cloned into pCR4®-TOPO (Invitrogen, Grand Island, New York). A total of 120 clones from each cell line and blood sample were isolated and sequenced to analyze predominant and rare variants. For comparison, variants from *A. marginale* VA passage 46 in ISE6 cells (the passage used as starting inoculum for cultivation in mammalian cells) were analyzed the same way. Thirty clones were analyzed from this sample which provided >90% probability of detecting variants present in the population at greater than 5% [6]. Nucleotide sequences were analyzed and translated into amino acid sequences using MacVector 10.6 (MacVector Inc., Cary, North Carolina) and Sequencher 4.8 (Gene Codes Corporation, Ann Arbor, Michigan). The hypervriable regions (HVR) of the amino acid sequences were aligned and compared using ClustalW [30]. The predominant variant of each cell line was determined as described in Materials and Methods S1.

Additional, *msp2* sequences cloned by several investigators from ticks, tick cells, and blood samples from acute and persistently infected animals infected with *A. marginale* VA, South Idaho, or Oklahoma strains [13], [17], [31] were compared with the sequences recovered in this study. The sequences of the *msp2* donor alleles of *A. marginale* VA and South Idaho strains [32] were included in the phylogenetic analysis of all the variants. Variant amino acid sequences were trimmed to contain only the HVR sequence and subjected to phylogenetic analysis using the total number of differences in MEGA 4.0 and Neighbor-Joining with 3000 bootstrap replicates to examine the relationship of variants to each other.

### Determination of msp2 donor allele repertoires

Genomic DNA was extracted from *A. marginale* VA infected blood using the Gentra Puregene DNA isolation kit (Qiagen). As previous studies have indicated that the *A. marginale msp2* allelic repertoires are positionally conserved between strains [33], we employed a locus specific PCR strategy using eight locus specific primer sets designated: P1, ψ1, ψ2, G11, 3H1, E6F7, 9H1, and TTV106 (Table 3). The PCR Master kit (Roche, Indianapolis, Indiana) was used as follows: 30 cycles of melting at 94°C for 30 s, variable annealing temperature for 30 s, and extension at 72°C (annealing temperatures and extension times are shown in Table 3). The PCR amplicons were ligated into the pCR4- TOPO vector using the TOPO-TA cloning kit (Invitrogen, Grand Island, New York), and then transformed into TOP10 *E. coli* cells. DNA was extracted by using the Wizard plus SV Miniprep DNA purification system (Promega, Madison, Wisconsin). The plasmid DNA was sequenced with the Big Dye kit (Applied Biosystems, Carlsbad, California). Inserts for the P1, G11, 9H1, and TTV106 loci were sequenced using HV univ for and HV univ rev primers [34], and 3H1, E6F7, ψ1, and ψ2 were sequenced using T3 and T7 primers. The *A. marginale* VA donor alleles have been deposited in GenBank with accession numbers VaP1: JN703159, VaG11: JN703155, Va9H1: JN703157, Va3H1: JN703158, VaE6F7: JN703156, Vaψ1: JN703161, and Vaψ2: JN703160 and VaTTV106: JN703154.

**Table 3:** Primers used for the amplification of *A. marginale* pseudogenes.

msp2 pseudogene	Primer sequence	Annealing temp (°C)	Extension (mins)
P1	P1 F1 5′- GTGGTTCCTGGGGTACATCTAGTATAGG-3′	60	4
	P1 Rx 5′- CTAGTCGCTGTATCATCAGCTTCAGTAC-3′		
G11	G11 Fx 5′- GCGACCAAACACAGCACATCCG-3′	60	4
	G11 Rx 5′- CAGAGCGGCGTTGCCTTGTC- 3′		
3H1	3H1 Fx 5′- CAGTCTCTTGTACCTCAACACC- 3′	57	2
	3H1 Rx 5′- CTTGGTAGCTGTATCGTCAGC- 3′		
E6F7	E6F7 Fx 5′- GGCCGGCAAGGTCAGCATAAGCTGTGG- 3′	57	2
	E6F7 Rx 5′- CTCGTATGACTGGCACAGTCAAACTTAC- 3′		
ψ1	ψ1 Fx 5′– CACCACCACTGCTACCAGTC– 3′	58	1
	ψ1 Rx 5′– GCCCAATCTGCTACCACCTCTG– 3′		
ψ2	ψ2 Fx 5′– CTTGGCCTACCAAATCTCGACCCTG– 3′	58	1
	ψ2 Rx 5′– CCCAGTTCCCACTTACCAGCACC– 3′		
9H1	9H1 Fx 5′- GCTCGCCAATCAACAACGTGTTCAC- 3′	57	2
	9H1 Rx 5′- CGCATCTTTGCCAATCTGGGTATTCC- 3′		
TTV106	TTV106 Fx 5′- GACGCATATACCTTGGCTCACCGTTC- 3′	58	2.5
	TTV106 Rx 5′- GTGCAGCACTACGGTGTTGGTGTG C- 3′		

### Bioinformatic and complexity analysis of Msp2 variants

Several parameters including charge, amino acid composition, and grand average of hydropathicity (GRAVY) were analyzed using ProtParam from the ExPASy proteomics server (www.expasy.ch/tools/protparam.html). The secondary structure of the variants was predicted using Psipred (http://bioinf.cs.ucl.ac.uk/psipred/) [35] and the Chou-Fasman method from MacVector®. The differences in structure between variants and their frequency was analysed to determine if patterns in variation coincided with changes in the potential secondary structure.

Additionally, complexity scores and donor allele usage for each of the cell lines and blood samples were determined [20], [29]. Preferential donor allele usage was evaluated by determining the frequency of recombination of whole or segments of specific donor alleles during production of new variants [20]. We additionally calculated the differences in donor allele usage within a population by comparing the predominance of all the variants containing complete or partial sequences of a particular donor allele's HVR. The predominance of the variants was calculated as described in the section *Cloning and analysis of msp2 variants expressed in vitro and in vivo* and in Materials and Methods S1. Significance was evaluated using chi-square.

### Analysis of differences in msp2 expression by qRT-PCR

RNA from *A. marginale* VA grown in ISE6, DAE100T, and RF/6A cells was extracted using the Absolutely RNA miniprep kit (Stratagene, La Jolla, California) and treated with Turbo DNA-free DNase (Ambion, Austin, Texas) to eliminate any remaining DNA. qRT-PCR was performed using primers Msp2f and Msp2r (Table 2), that are complementary to the conserved flanking regions of *msp2* at positions 4798–4817 bp and 5163–5144, respectively, of the *A. marginale* VA *msp2* operon GenBank accession number: AY132312). *msp2* expression was normalized against expression of RNA polymerase (*rpoB*) and 16 s rRNA genes using the comparative Ct method [36]. All reactions were performed in a Stratagene Mx3005 QPCR machine using the Brilliant II SYBR green QRT-PCR Master Mix 1-Step kit (Stratagene, La Jolla, California) with a primer concentration of 200 nM, 30 nM ROX (as reference dye), and 100 ng of total RNA. Cycling conditions were as follow: an initial step of reverse transcription for 30 min at 50°C, followed by a denaturing step of 10 min at 95°C, and 40 cycles with denaturing at 95°C for 30 seconds, an annealing temperature of 63°C for 1 min, and extension at 72°C for 1 min, with a final denaturing step at 95°C and annealing for 30 seconds at 63°C to determine the dissociation curve. Statistical significance of gene expression results was evaluated using REST© 2008 [37] (http://www.gene-quantification.de/rest-2008.html), with 2000 randomizations.

### Accession numbers for Msp2 variants

The predominant *msp2* variant from tick cell lines was designated as V1 (EU496530), whereas the predominant variant from mammalian cells was designated as V2 (EU496531), the *msp2* variant shared among tick cell lines only was designated V4 (EU496533), and variants shared between the mammalian and tick systems were designated V3 (EU496532) and V5–V12 (JQ082310, EU512243, EU496534, EU517681, EU499673, JQ177150, EU499670). Variants shared between blood samples were designated V13 (EU627153) and V14 (EU627154). *msp2* variants unique to the tick cell line ISE6 were assigned codes V15 to V17 (EU496539, JQ082308, JQ082309). Variants unique to BME26, DAE100T and IDE12 cells were coded V18 to V21 (EU496538, EU496536, JQ082306, JQ082307), V22 to V26 (JQ177151–JQ177155), and V27 to V31 (EU499671, EU499672, JQ177156, JQ177157) respectively. Variants unique to the mammalian cell lines Vero and RF/6A were coded V32 to V36 (EU512244–EU512248), and V38 to V41 (EU517678, EU517679, EU512237, EU512238), respectively. *msp2* variants from PA344 were assigned codes V42 to V74 (EU517683–EU517694, EU526866–EU526882), and variants from PA291 were given the codes V75 to V97 (EU526883–EU526897, EU627148–EU627155). GenBank accession numbers are given in parentheses.

## Results

### A. marginale development in the different cell lines

**Figure 1:**
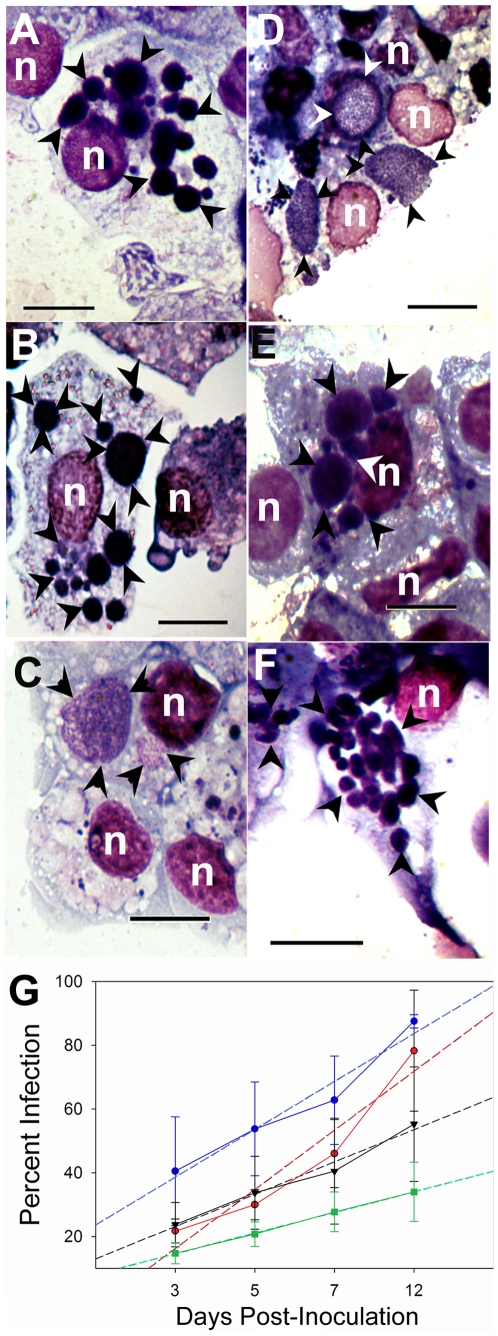
Differences in colony morphology and percentages of infection during in vitro replication in cell lines. Giemsa stained preparations show the colony morphology of *A. marginale* VA in (A) Vero cells, (B) DAE100T cells, (C) BME26 cells, (D) ISE6 cells, (E) RF/6A cells, and (F) IDE12 cells. Arrows point to the *A. marginale* VA colonies growing in each cell line. Cell nuclei stained in lighter red-purple color and marked with the letter n. Bar = 20 µm. (G) Increase in the percent of infected tick and mammalian cells during a 12-day period. The solid red line with red circles represent the infection rate of ISE6 cells at each time point. The solid blue line and blue circles represent IDE12. Solid black line and triangles represent to the infection rates in BME26 cells, whereas the green squares and line represent to the values for the infection rates in Vero cells. The linear regression calculated from the percent infected cells over time is shown as a dotted line in the color corresponding to the cell line represented.Bars represent the standard deviation for each time point.

*A. marginale* VA invaded and infected all the tick and mammalian cell lines, replicating in intracellular inclusions as previously reported [21], [28], [38]. Infection rates of *A. marginale* VA in each of the cell lines differed significally (P<0.001). Infection rates in *I. scapularis* cell lines were higher (6.4% and 5.1% increase in infected cells per day) compared to the other tick cell lines (4.9%–3.4%) (Table 4 and Figure 1G). In both *I. scapularis* cell lines, >90% of the cells were infected by 7 to 12 days after inoculation, and growth was slowest in mammalian cell lines, while infection rates were intermediate in *R. microplus* and *D. andersoni* cell lines (Table 4; Figure 1G). In mammalian cells *A. marginale* grew more slowly than in tick cells (Table 4; Figure 1G) and approximately 3 weeks elapsed before 80 to >90% infection was reached. These results may mirror the natural behavior of *A. marginale*, which spreads quickly through tick tissues, but develops slowly in cattle. Male *D. andersoni* were infectious one week following acquisition feeding, while calves developed anaplasmosis 3.5 weeks later [39]. The closely related *A. phagocytophilum* likewise requires long periods of adaptation when transferred from tick to mammalian cell cultures. The transitional period is accompanied by recombination of different *msp2/p44* paralogs into the expression locus, resulting in a heterologous population of bacteria that display predominance of certain variants in a manner similar to that described here for *A. marginale* VA [11].

**Table 4:** Growth rates differences during infection of various tick and mammalian cell lines.

Cell line	Infection rate	R value	P value
	(IC/day*)		
ISE6	6.441	0.908	<0.001
IDE12	5.12	0.83	<0.001
BME26	3.398	0.683	0.004
DAE100T	4.877	0.724	0.002
Vero	2.096	0.78	<0.001
RF/6A	3.102	0.887	<0.001

* IC/day = Infected cells increase per day.

Light microscopic examination of infected cells showed that colony morphology also varied greatly from cell line to cell line. In both mammalian cell lines *A. marginale* VA developed into several small colonies that filled most of the cell (Figure 1A and 1E). In DAE100T and IDE12 cells the bacteria similarly produced four or more small colonies (Figure 1B and 1F, respectively), whereas in BME26 and ISE6 cells contained one to three large colonies (Figure 1C and 1D, respectively).

### Msp2 expression patterns

**Figure 2:**
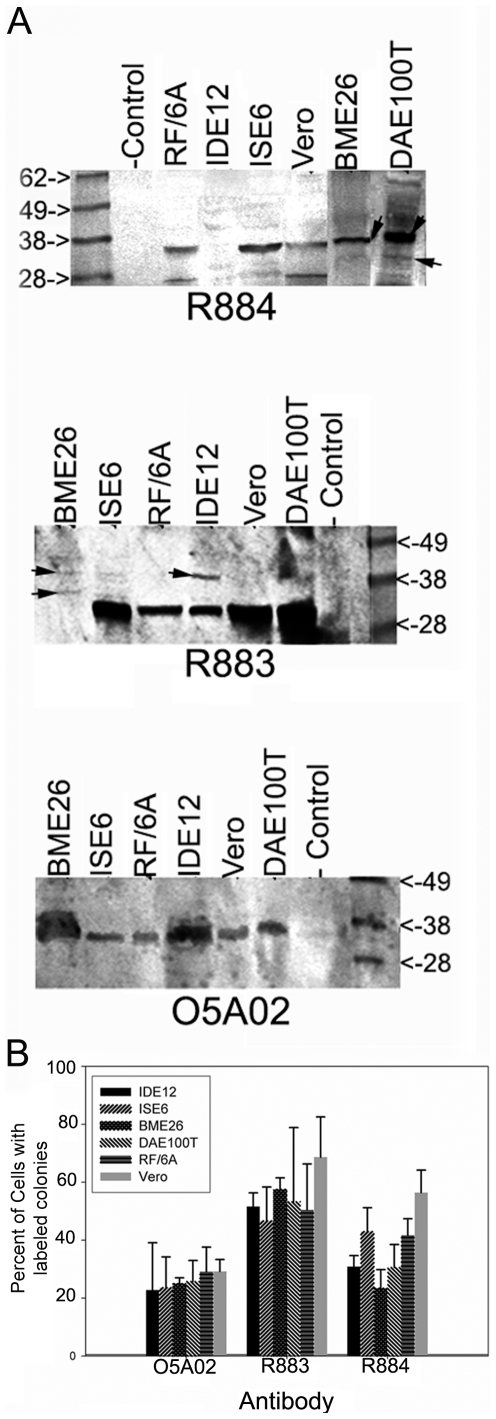
Changes in the expression of Msp2 variants during infection of tick and mammalian cells. (A) Detection of Msp2 variants with monospecific antibodies R884 and R883 and monoclonal antibody O5A02 by Western blots. Controls consisted of uninfected ISE6 and IDE12 cell protein extracts. (B) Variation in recognition between Msp2 specific antibodies using immuno fluorescence assays. Bars length represent the percentage of *A. marginale* VA colonies recognized by a monoclonal antibody or monospecific antibodies in a specific cell line. Error bars indicate the standard deviation.

Western blots probed with different monoclonal and monospecific antibodies revealed variability in the molecular weight of Msp2s expressed during growth of *A. marginale* VA in the different cell lines. The most prominent bands spanned a range of 36 to 44 kDa, which is consistent with the molecular weight previously reported for Msp2 [40]. Blots probed with monoclonal (mAb) and monospecific antibodies (msAb) identified several Msp2 variants expressed in different cell lines. In *A. marginale* VA proteins from BME26 cultures the Msp2 msAb R883 detected two small bands, whereas a blot of the same sample when probed with mAb O50A2 showed a single prominent band of different molecular weight (Figure 2A). *A. marginale* VA replicating in BME26 cells lacked expression of Msp2 variants that are recognized by these antibodies. However, R883 msAb detected two minor variants in the BME26 sample, one of which was similar in size to a minor variant detected in the population grown in IDE12. R884 msAb detected different sized variants in BME26 and DAE100T and an additional variant not detected in other samples (Figure 2A).

Analysis of Msp2 variant expression in *A. marginale* VA colonies by IFA revealed a cell type dependent pattern. Statistical analysis demonstrated significant differences in the ability of antibodies to recognize colonies in cell lines (P = 0.023) as well as differences in the percentage of colonies labelled by different antibodies in a specific cell line (P<0.001) (Figure 2B). The greatest differences were apparent in the percentage of colonies in different cell lines that were recognized by a particular antibody. These monoclonal and mono-specific antibodies likely bind to a specific variant or different set of variants, respectively expressed in the cell lines. R884 msAb labelled to up to 60% of the colonies in Vero cells compared to around 25% in BME26 and 30% in IDE12 and DAE100T (Figure 2B). Similarly, R883 showed significant variability with around 75% of the colonies recognized in Vero cells *versus* only 50% in ISE6. In contrast, mAb O5A02 recognized 5–15% more colonies in mammalian than in tick cells, depending on the cell line, however, these differences were not statistically significant (Figure 2B).

### Expression of Msp2 variants during infection of different cell types

**Figure 3:**
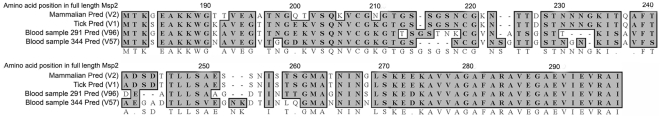
Amino acid sequence alignment of predominant variants found in culture systems and blood samples. Regions that are boxed and shaded in grey indicate conservative amino acid sequences. Conserved amino acid changes are identified by a period. Gaps represent sites of insertions and deletions. The letters in the bottom line represent the amino acid sequence of the consensus sequence. Numbers indicate the position of the amino acids relative to the full length expression locus sequence. V1 represents the predominant variant in tick cell cultures after 21 passages in BME26, 20 passages in DAE100T, 27 passages in ISE6, and 28 passages in IDE12 (following inoculation with cell-free bacteria). V2 corresponds to the predominant variant of tick cells at the following passages: 20 passages in Vero and 19 passages in RF/6A (following inoculation with cell-free bacteria). All samples were taken when infections levels reached >90% at subculture. Average culture times in each cell line were 7 days in ISE6 cells, 9 days in IDE12 cells, 12 days in DAE100T cells, 16 days in BME26 cells, 18 days in RF/6A cells, and 21 days in Vero cells.

**Figure 4:**
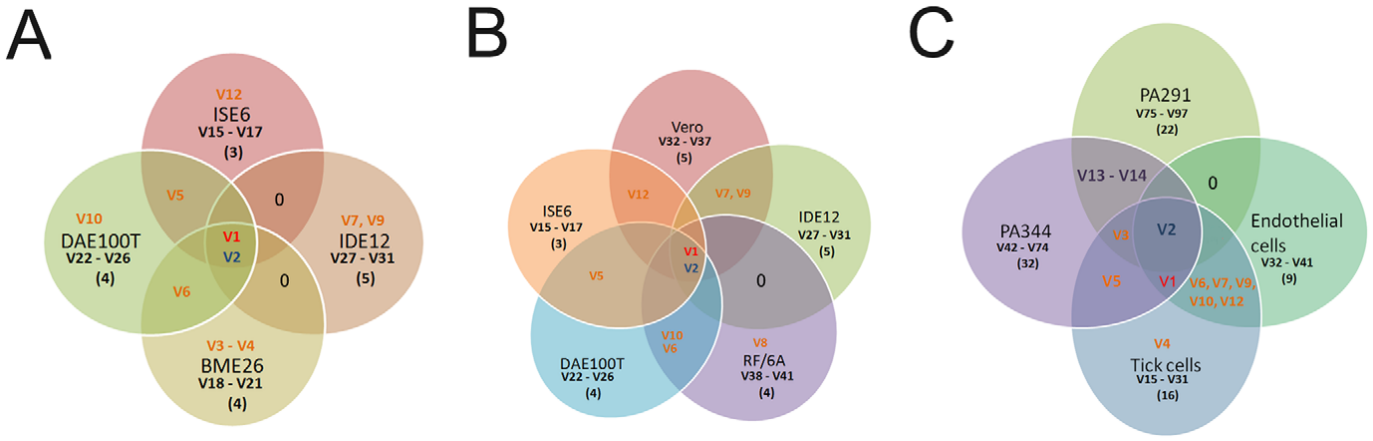
Generation of unique and shared variants during infection of blood and cell lines. Venn diagrams showing the relationship between variants identified in cell lines and blood samples during the present study. Orange lettering identifies variants shared between mammalian and tick cell lines, and infected bovine blood. Black lettering identifies variants that are unique to the respective cell line or blood sample, and includes the number of variants in parentheses. Black zeros (0) represent areas of potential overlap where no shared variants were identified. V1 (in red) is the tick predominant variant and V2 (in blue) is the mammalian predominant variant. A) Unique and shared variants in tick cell lines, B) Unique and shared variants in tick and mammalian cell lines, and C) unique and shared variants in the tick and mammalian cell cultures, and the two blood samples (PA291 and PA344).

Clones harbouring the sequence amplified from the *msp2* expression locus in each cell line were sequenced and translated into amino acid (aa) sequences for molecular analysis of Msp2 variation. The HVR portions (180–290 aa) of Msp2 [7] from all clones were aligned and the number of clones of the total 120 clones analyzed for each cell line and blood sample that presented an identical sequence was used to estimate the predominance of a variant in the mixed population. An alignment of aa sequences in variants predominating in mammalian (V2) and tick (V1) cell cultures and blood from two infected animals (V96 in PA291, V57 in PA344) is shown in Figure 3. Changes in the constant regions were used to estimate sequencing errors [13], and only those HVRs with <97% identity were classified as unique. All tick cell lines shared the same predominant variant (designated V1) that was present in 86%, 76%, 73%, and 68% of the clones from ISE6, BME26, IDE12, and DAE100T, respectively (Figure 4A). This same variant (V1) was also recovered from the original inoculum (*A. marginale* VA passage 46) and was the predominant variant before inoculation into tick and mammalian cell lines. V1 was also recovered from mammalian cells but with lower frequency (8% from RF/6A and 13% from Vero cells) (Figure 4B). V1 differed by 9 aa changes from the predominant variant found in mammalian cells (V2), which corresponds to a segmental change: the V2 HVR is identical to donor allele VaE6F7, and V1 contains a segment from Vaψ2 recombined onto this template (Figure 3). V2 was present in blood, tick and mammalian cells (Figure 4A–C), representing 83% and 70% of the clones recovered from RF/6A and Vero cells, respectively, but only 4% to 11% of the clones recovered from populations in the tick cell lines.

Variants V1 and V2 differed from the predominant variants found in the blood samples PA291 and PA344 by 29 to 33 aa changes, respectively (Figure 3). Interestingly, V2 was present in both PA291 and PA344 blood samples (Figure 4C), whereas V1 was only found in the sample from Hereford calf PA344 that had been inoculated with *A. marginale* VA grown in IDE8 tick cells, albeit at low frequency (3.3%) (Figure 4C). The presence of V2 in both samples indicates that this variant may be more suited for the mammalian environment, whereas the lack of V1 in PA291 could reflect that this animal was inoculated with infected blood and thus this variant was probably absent from the inoculum. None of the predominant variants found in the blood samples (V57 and V96) were present in the tick or mammalian cell cultures, suggesting they were generated through immune pressure, and only blood variants with minor frequency (V8 and V3) were shared with some but not all of the cell lines (Figures 4C).

The number of *A. marginale* VA Msp2 variants differed in each cell line and blood sample. The greatest number of variants was found in the blood from the acutely infected animal PA344 (38 variants), followed by the blood sample from PA291 from which we obtained 26 variants (Figure 4C). Variation during culture was highly reduced and the total number of variants present in tick cells differed among cell lines (7–11 variants) (Figure 4A). *A. marginale* VA from *I. scapularis* cell lines supported the fewest variants, i.e., there were only seven variants in ISE6 and eight in IDE12, whereas BME26 had nine and DAE100T cells presented ten (Figure 4A). By comparison, we detected 11 variants in Vero and 10 in RF/6A cell lines (Figure 4B). Interestingly, many of the minor variants found in each cell line were unique to that specific line (Figure 4A–B). The number of unique variants was highest in the two blood samples, PA291 (22) and PA344 (32) (Figure 4C), but new variants were also generated during *in vitro* culture in the absence of immune pressure. Bacteria growing in tick cell lines produced a total of 15 unique variants (Figure 4C) with the IDE12 cell line showing the highest number of unique variants (5) (Figure 4A). On the other hand, mammalian cells presented 9 unique variants, with 5 unique variants in Vero cells and 4 in RF/6A cells (Figure 4B).

### Analysis of variation in vivo and in vitro

In order to place our variants in the context of *msp2* donor alleles and expressed variants from other sources, we undertook a phylogenetic analysis that included all *A. marginale* variant and pseudogene (donor allele) sequences in GenBank together with those obtained during this study. These comprised 14 tick and 13 blood variants from *A. marginale* VA, South Idaho, and Oklahoma strains deposited in GenBank, sequences from pseudogenes of the sequenced genomes of *A. marginale* VA and South Idaho, and all variants obtained during this study (Figure S1). One of the larger clades (marked in the figure by the light green oval) contained the mammalian cell culture (V2) and the tick cell culture predominant (V1) variants, which fell into separate smaller groups. V1 grouped with two other tick cell culture variants, while V2 associated with variants from ticks and tick cell culture, cattle, and one of the *A. marginale* VA pseudogenes. Although the two largest clades both included variants from all sources analysed, the great majority of tick and tick cell culture variants segregated with the clade containing V1 and V2. 71% (10 out of 14) of the variants generated during *in vivo* infection of ticks fell into this clade, and one tick cell culture variant (V30) was an outlier unrelated to any others (Figure S1).

Similarly, the mammalian variants obtained during culture in endothelial and Vero cells also grouped primarily in the major clade containing most tick variants, but did not show preferential association with variants from any one source (Supplemental Figure S1). Blood variants were most closely related to other blood variants, but some were found dispersed throughout the clade dominated by tick variants. Thus, the phylogram divides variants into two major classes: those arising under immune selection, showing large changes, and the others evolving without it, showing small changes, as reflected in branch length. All except two (VTTV106/VaE6F7 and VaP1) of the donor alleles were positioned throughout the clade dominated by blood variants (Figure S1).

### Influence of host cell type on donor allele usage and variant complexity

Complete genome sequencing of the St. Maries and Florida strains reveals seven or eight *msp2* pseudogenes, and that the *msp2* loci are syntenic for the two strains [41]. Using these genome sequences, a set of locus specific PCR primers were developed (Table 3). Testing these primers on the fully sequenced St. Maries strain did not produce artifactual chimeras. Southern analysis indicated that *A. marginale* VA contained eight pseudogenes for *msp2* (Figure S2). The donor allele repertoire was determined for *A. marginale* VA using the locus specific primers and sequencing. Examination of the *A. marginale* VA repertoire revealed that the pseudogene HVRs in the loci designated VaE6F7 and Va1were identical to the sequences in these two loci in both the St. Maries and Florida strains, while the pseudogenes in the loci designated Vaψ2, Va9H1, VaTTV106 were identical to sequences in the Florida strain at the syntenic loci. Three novel *msp2* HVR pseudogene sequences were obtained at loci VaP1, VaG11 and Va3H1.

**Figure 5:**
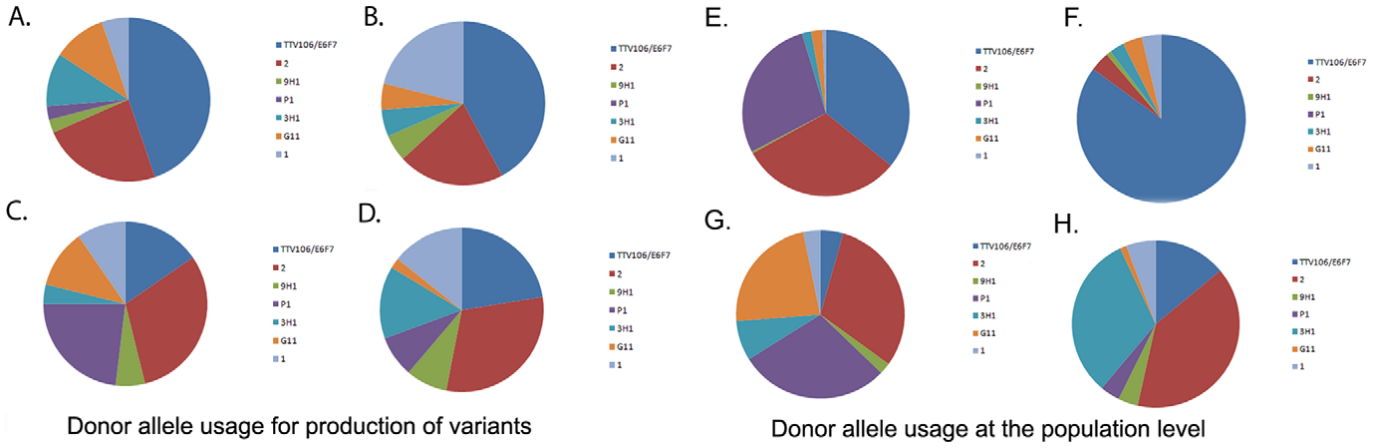
Donor allele usage in Msp2 variant production by *A. marginale* during growth in vivo and in vitro. Each color represents the usage of one of seven specific donor alleles (i.e. pseudogenes) during infection of a particular cell culture system, and during acute or chronic infection in cattle. Allele usage was assessed to determine its preferential use for generation of new variants (5 A–D) and to determine its overall representation among all variants in a population (5 E–H). The frequency of use of each allele in the production of variants was calculated from the total number of variants that contained sequences from a specific donor allele divided by the total number of variants. This represented the preferential usage of an allele for the production of variants and is shown in (A), for tick cell cultures; (B), mammalian cell cultures, C), acutely infected bovine PA344; and (D), chronically infected bovine PA291. Overall allele usage in a population was calculated from the total number of clones that comprised DNA sequence from any specific allele, divided by the total number of clones recovered from each system. This provided an estimate of the use of an allele in any variant, whether major or minor, recovered from a population. (E), Overall usage of variants in tick cell cultures; (F), in mammalian cell cultures; (G), in the acutely infected bovine PA344; and (H), in the chronically infected bovine 291. As an example, comparison of corresponding pie charts A and E demonstrates that allele P1 is infrequently found in variants from tick cell cultures, although it is recovered in a large number of clones. Whereas, a comparison of pie charts B and F shows that allele TTV106/E6F7 is recovered from mammalian cell cultures about as often as alleles 2 and 1, although segments of TTV106/E6F7 are recovered in the majority of different clones.

Analysis of donor allele usage in populations from different cell types showed preference for the expression of certain donor alleles within a population (chi-square value = 451.75; p≤0.001). Similarly, generation of variants during infection of any of the host systems was not random (chi-square value = 188.02; p≤0.001). Va TTV106/E6F7 and Va ψ2 were highly used during generation of variants *in vitro*, being present in 94% and 50%, respectively, of HVRs in *A. marginale* from tick cells, and in 89% and 44% of *A. marginale* growing in mammalian cells. However Va ψ1 was used preferentially during generation of variants in mammalian cells, either by recombination of all or only portions of the pseudgene HVR, with 44% usage compared to only 11% in tick cells (Figure 5A and 5B). Va TTV106/E6F7, Va ψ2, and Va P1 donor alleles were preferentially used in the tick systems with 97%, 87%, and 78% representation, whereas only Va TTV106/E6F7 was predominant in the mammalian cells system 91% (Figure 5E and 5F).

We observed differences in the representation of donor alleles in Msp2 variants from PA344 and PA291 blood. *A. marginale* VA Msp2 variants in blood from the acutely infected animal PA344 used Vaψ2 and VaP1 somewhat more frequently (64% and 48%, respectively) than those from PA291 (Figure 5C). Donor allele VaG11 was highly used (42%) in PA344 populations (Figure 5G), while 29% and 20%, respectively, of variants expressed in both blood samples contained Vaψ1 sequences. In the chronically infected animal PA291, 30% of variants comprised Va3H1 and were represented at 50% in the population, while VaP1 sequences were only found in 6% of the population at 17% frequency of use in expressed variants (Figure 5D and 5H). As in PA344 blood, Vaψ2 was present in 62% of variants in the population and contributed 62% of variant sequences (Figure 5D and 5H). By contrast, *A. marginale* St. Maries predominantly utilized *msp2*ψ1 for generation of blood variants [20], suggesting that genetic background of an isolate may influence pseudogene utilization. Va9H1 was under-represented in all populations independent of the source and whether comparing usage at the population level or during generation of individual variants (Figure 5A–H), and this finding is similar to that previously reported [20].

Variants from both blood samples had the highest complexity scores at 1.5 (PA291) and 1.13 (PA344) (Figure S3), followed by variants from mammalian cell cultures (1.1), and the tick cell cultures presented the lowest complexity score (0.93) (Figure S3). The low average scores in PA344 and PA291 (both of which were splemectomized) and the cell cultures are due to the presence of several simple variants in these samples.

### Bioinformatic analysis of variant characteristics

**Figure 6:**
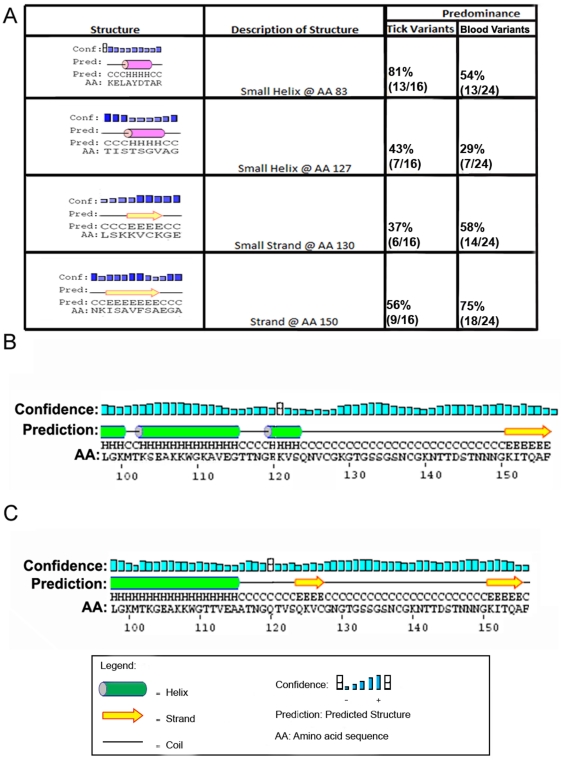
Changes in putative secondary structures of predominant *A. marginale* VA Msp2 variants in vivo and in vitro. (A) Changes in the putative structure of Msp2 variants produced during *in vivo* infection of ticks and cattle. The percentages represent the predominance of the structure in all the variants found either in experimentally infected ticks or cattle. Numbers in the parenthesis indicate the number of variants presenting the structure divided by the total number of variants analyzed in that specific system. (B) Predicted structure of the hypervariable region of Msp2 that predominates during infection of tick cell lines. (C) Predicted structure of the hypervariable region of Msp2 that predominates during infection of mammalian cell lines. Symbols representing protein structure and the confidence value attributed to the accuracy of the predicted structure are explained in the legend box.

Analysis of pI, molecular weight, charge, and hydropathicity of the amino acid sequences of cell culture variants revealed no correlation with host cell type. However, analysis of the predicted secondary structure of the amino acid sequences using Psipred identified a change in the secondary structure in Msp2 from *A. marginale* VA growing in tick cell lines when compared to the most frequent structure found in mammalian cell lines and blood. Psipred and Chou-Fasman analyses predicted that the changes in amino acid sequence at position 195 to 206 (relative to full length Msp2 of *A. marginale* VA)from GTTNGEKVSQNV (in the tick cell culture predominant variant) to AATNGQTVSQKV (in the mammalian cell culture predominant variant) result in a modification of the secondary structure of the protein in this region (Figure 6B and C). Other amino acid changes occurred in the sequences of these two proteins, however they did not lead to modifications in the putative structure of the variants when compared with one another (Figure 3). The sequence GTTNGEKVSQNV in the tick cell culture predominant variant was predicted to produce an α- helix, whereas AATNGQTVSQKV in the mammalian cell culture predominant variant would form a β-strand. The α-helix structure was present in 44% of the variants obtained from tick cell-grown *A. marginale* VA, while around 57% of the variants from mammalian cell cultures presented the putative β-strand structure.

Similarly, analysis of variants generated *in vivo* detected structures that were more commonly expressed in either ticks or cattle. We identified a β-strand at the sequence KISAVFSA that was more common in blood variants (75%) than in tick variants (56%), and sequence SKKVCKG was found in 58% of the blood variants but in only 37% of tick variants (Figure 6A). The sequence KELAYDTAR, generating a small helix in the HVR was present in 81% of tick variants compared to 54% of the bovine blood variants. Similarly, 43% of tick variants contained the helix sequence STSGVA that appeared in only 29% of the variants from cattle (Figure 6A). An alignment of the aa sequences of the HVR in the predominant variants from cell cultures and bovine blood (Figure 3, this study) revealed additional conservative and non-conservative aa changes elsewhere (Figure 3). However, these were not predicted to cause modifications in the putative structure of the variants.

### Relative expression of msp2

**Figure 7:**
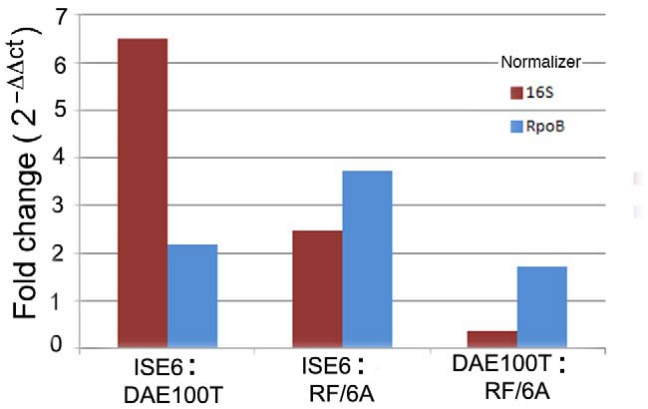
Relative expression of msp2 during infection of tick and mammalian cell lines. Bar length represents the fold change in expression of *msp2* during A. marginale VA infection of ISE6 vs DAE100T, ISE6 vs RF/6A, DAE100T vs RF/6A, using *rpoB* (bars in blue) and *16s rRNA* (bars in red) as normalizers. Relative expression was calculated using the comparative Ct method (2^−ΔΔCt^) [36]. Bars represent the fold values calculated with the mean Ct values.

In order to determine whether the qualitative changes in *msp2* expression were accompanied by quantitative changes, we determined levels of expression of all *msp2* transcripts in two tick cell lines and one mammalian cell line (Figure 7). Data reveal greatest transcriptional activity of *msp2* in *A. marginale* VA growing in ISE6 tick cells, and least in RF/6A rhesus cells, while *msp2* transcription was intermediate in DAE100T cells from *D. andersoni* ticks. *msp2* expression during growth of *A. marginale* VA in ISE6 cells, when normalized against 16 s yielded a fold change of ∼6 over expression in DAE100T cells (P(H1) = 0.602), whereas when normalized against *rpoB*, fold change less pronounced. *msp2* transcript abundance in ISE6 was about twice that in RF/6A cells, while the difference between DAE100T and RF/6A was negligible (P(H1) = 0.181) (Figure 7). Differences in the roles (and therefore the stability and turnover) of 16 s rRNA which serves a structural role in the ribosome, and of mRNA for *rpoB*, likely are responsible for the seeming differences in *msp2* expression when each is used as a normalizer for that gene. The statistical significance of the analysis was affected by the variation between samples, possibly due to the use of asynchronous cultures.

## Discussion

In this study, we took advantage of the great diversity of cell lines that support replication of *A. marginale* VA in order to examine the propensity of this pathogen to undergo variation of a major surface protein, Msp2, in response to changing host cell environments [8], [13]–[16]. We utilized the original culture isolate of *A. marginale* VA [21] because it grows in a wide range of tick cell lines derived from natural and experimental vectors [38], [42]. In addition, *A. marginale* VA readily infects and grows in at least two mammalian cell lines as well as in primary cultures of bovine vascular endothelial cells [28], [43]. We studied the expression of Msp2 variants of *A. marginale* VA in order to understand the molecular mechanisms that underlie antigenic variation in this organism during its residence in tick and mammalian host cells. We demonstrated generation of predominant Msp2 variants that differed in amino acid sequences in populations growing in specific cell environments. Populations growing in tick cells presented predominantly V1, a sequence that does not correspond to a donor allele, demonstrating that the tick-adapted variant has not been retained in the donor allele repertoire. The populations growing in endothelial cells presented predominantly V2. In an independent study, an *A. marginale* VA variant identical to V2 also predominated in RF/6A cells as well as in bovine testicular vein endothelial cell cultures [43], further evidence that this variant was not randomly generated in our endothelial cell cultures.

Research with the related pathogen *A. phagocytophilum* that infects neutrophil granulocytes similarly identified profound differences in expression of Msp2 variants while residing in tick *versus* mammalian cells. Unlike *A. marginale*, the genome of *A. phagocytophilum* encodes over 100 *msp2/p44* pseudogenes that are recombined into the expression locus intact, without recombination of the HVRs [11], [44]. Nonetheless, *A. phagocytophilum* expression of variants changed so that major (or predominant) variants of P44 became minor (or less frequent) variants following transfer from mammalian hosts to ticks, or to 24°C [45], in a manner reminiscent of antigenic variation in *A. marginale*. While temperature likely is one of the factors regulating *p44* expression, other, as yet unknown biological cues derived from host and vector tissues may induce changes in the composition of the Msp2/P44 pool expressed by *Anaplasma* species [45], [46]. In infected ticks, the repertoire of *A. marginale* variants became restricted compared to that in the blood meal source [12], [31]. Some variants expressed in the salivary glands were identical to those in the blood stabilate used to infect the cattle upon which the ticks had fed, but new variants were formed as well [12], [31]. In general, tick variants were most closely related to each other, and the same was true for mammalian variants, regardless of whether variants were generated *in vivo* or *in vitro* (Figure S1). Because antibodies to *A. marginale* were absent from our cell cultures, selection of Msp2 variants should reflect the environment provided by the specific cell line. Thus, nearly twice as many unique variants were detected in the four tick cell lines as in the two mammalian cell lines.

Each cell line used in this study presented differences in morphology and biological properties that possibly affected bacterial invasion and replication. The absence of a functional innate immune system in ISE6 cells, which lacks phagocytic properties and does not produce immune peptides [47], might explain why it supported the fewest Msp2 variants. The extended period during which *A. marginale* VA was propagated continuously in ISE6 cells selected for the most suitable variants, as observed for *A. phagocytophilum* [11], and at the same time favoured growth in all tick cell lines through production of the shared predominant variant V1. IDE12 and BME26 cells produce immune peptides and are phagocytic [47], and DAE100T cells contain granules resembling those present in tick salivary gland acini (A. Palmer and T. Kurtti, unpublished results). These different environments likely drove variation of Msp2 in *A. marginale* VA, and similar mechanisms could facilitate invasion and exploitation of different hosts and tissues. The increased number of variants observed in the mammalian *versus* the tick cell lines could reflect their origin from primates, which are naturally resistant, but at the same time highlights the adaptive potential of *A. marginale*. Nonetheless, growth of *A. marginale* VA in primary bovine vascular endothelial cells generated the same dominant Msp2 variant as did growth in the rhesus microvascular endothelial cell line RF/6A and in Vero cells [43]. This result, together with evidence that *A. marginale* VA may infect bovine microvascular endothelium *in vivo* [48], validates the use of RF/6A cells to model *A. marginale* infection of bovine cells.

Based on a comparison with other gram-negative bacteria and characteristics such as outer membrane localization and predicted amphipathic and antiparallel ß-strand structures, it was suggested that Msp2/P44 of *A. phagocytophilum* acted as a porin [18]. The amino acid sequence GTTNGEKVSQNV (Figure 6 C) was predicted to result in formation of a β-strand (Chou-Fasman method and Psipred), and this sequence predominated during growth in mammalian cells. This sequence was not present during infection of tick cells indicated the corresponding structure was not required. Similarly, our analysis of variants *in vivo* demonstrated β-strands in bovines and helices primarily in ticks. An analysis of pseudogene usage in cattle infected with the St. Maries strain of *A. marginale* likewise revealed that an allele, *msp2ψ*1, encoding a β-strand, was significantly overrepresented [20], further supporting selection of these structures in the mammalian host.

Our results show that the composition of Msp2 variant populations differs among *A. marginale* VA from cattle, and tick *versus* mammalian cell lines (Figure 4). Although V1 and V2 are both present in cell lines and blood, V1 is dominant only in tick cells, and V2 only in mammalian cells, including bovine blood. In addition to dominant variants, each environment provokes generation of minor variants, creating unique profiles. Generation and selection of Msp2 variants is thought to be governed in part by the fitness they impart to the organism, with simple variants favored as long as immune pressure does not eliminate them [20]. We hypothesized that this should be reflected in the degree of *msp2* expression, and indeed, *msp2* transcripts were most abundant in ISE6 tick cells (Figure 7) in which variants also had the lowest complexity scores (Figure S3). When we compared donor allele usage during infection of bovine blood and the two *in vitro* systems (mammalian and tick cells), we found that certain donor alleles were preferentially used in populations to generate new variants. Three donor alleles in particular, i.e., VaTTV106/E6F7, VaP1, and Vaψ2, were the source of variants generated in ticks. This strengthens the hypothesis that certain donor alleles or variants provide a fitness advantage to the organism when infecting specific host systems, and consequently become predominant through selection [20].

This study further elucidates the molecular mechanisms that underlie antigenic variation in *A. marginale* as it cycles through ticks and mammals. The conformational changes induced in Msp2 in response to host cell environment offer insight into the role of this molecule beyond that in immune-evasion. In this regard, susceptible mammalian cell lines complement the use of tick cell lines. However, conclusions about the function of Msp2 should not be driven only by predictions of the secondary structure, and studies to determine the precise function of Msp2 are necessary.

### Conclusions

Our analysis of the development of *A. marginale* VA *in vitro* revealed complex changes following transfer from tick to mammalian cell lines similar to those that occur during its life cycle. *A. marginale* VA grew in two mammalian and four tick cell lines producing strikingly different colonies in each. The expression of specific Msp2 variants depended on the host cell type and resulted in changes in Msp2 detected by reactivity with specific antibodies. Molecular analysis showed predominant expression of Msp2 variant V1 in ticks and tick cell lines, whereas Msp2 variant V2 predominated in mammalian cell lines. Msp2 V1 differed in 9 amino acids from V2. Further, analysis of Msp2 variation during *in vitro* growth of *A. marginale* VA in tick and endothelial cells demonstrated the ability of the organism to generate unique variants present only in specific cell lines. Selection for variants presenting a specific secondary structure predicted by Psipred in the HVR of Msp2 during infection of cattle and ticks *in vivo* was supported by generation of the same structures in mammalian *versus* tick cell lines *in vitro*. Populations of *A. marginale* VA preferentially utilized certain *msp2* alleles for generation of Msp2 variants during development in tick and mammalian cell lines in a manner consistent with what was seen in ticks and cattle. The selection of bacteria presenting a specific structure in the Msp2 protein suggests that this protein plays an important role in the ability of *A. marginale* to infect and survive in divergent hosts. Relative expression of *msp2* did not significantly differ depending on the cell line in which *A. marginale* VA grew, as shown by qRT-PCR results. These findings underscore the putative divergent functions of Msp2 in the vector as opposed to the mammal, as well as the need to define the function of this protein.

## Supporting Information

Figure S1 **Phylogram of Msp** ***2*** **variants generated in vivo and in vitro.** Neighbor joining tree generated using the amino acid sequences of the Msp2 HVR from several variants obtained during infection of *Dermancentor spp.* ticks, bovine hosts, and tick and mammalian cell cultures with several strains of *A. marginale*. HVRs are designated by their GenBank Accession numbers, along with the HVR from variants obtained in this study, designated by their variant code (e.g. V1, V2, etc). Variants generated during infection of salivary glands of ticks *in vivo* or during *in vitro* culture of *A. marginale* in tick cell lines are presented in light green. Predominant tick variant (V1) is shown in dark green and indicated by a green arrow. Variants marked with an asterisk (*) represent GenBank Msp2 sequences from *A. marginale* VA. Variants produced during acute phases of bovine infection in erythrocytes are shown in dark blue and those generated later during the persistent phase, or chronic infection, are shown in light blue. Mammalian cell culture variants are shown in purple. V2 is shown in dark purple and indicated by a purple arrow. Donor allele sequences are marked in red letters. Variants shared between the tick and mammalian cell cultures are shown in orange. Branch lengths show the number of amino acid changes between the variants. Values next to branches correspond to 3000 bootstrap analyses using MEGA 4.0. Only branches with over 50 percentage support are indicated in the tree. Vertical bar in lower right represents five amino acid changes.
(TIF)

Figure S2 **Genomic Southern analysis of** ***msp2*** **.** Lane M contains labeled lambda-HindIII markers with sizes (in kbp) indicated to the left, lanes StM and Va contain FspI digested genomic DNA from the St. Maries and *A. marginale* VA, respectively. The expression site is indicated (ES).
(TIF)

Figure S3 **Changes in Msp2 complexity scores during infection of four different systems.** Complexity was measured by determining the number of expression site segments derived from one of the eight different donor *msp2* alleles encoded in the genome of *A. marginale* VA. Bars represent the average of the complexity score in a chronically infected animal (PA291), an acutely infected animal (PA344), and during culture in tick and mammalian cells. Lines represent the standard deviation of the mean. “n" stands for the number of variants present in each population that was used to calculate complexity scores.
(TIF)

Materials and Methods S1 (DOC)

File S1 **Abstract in Spanish.**
(DOC)
