# Supplementary figures and images for: Expression Patterns of Anaplasma marginale msp2 Variants Change in Response to Growth in Cattle, and Tick Cells versus Mammalian Cells
Source: PLoS One. 2012 Apr 25;7(4):e36012. doi: 10.1371/journal.pone.0036012 (PMC3338850; doi:10.1371/journal.pone.0036012)

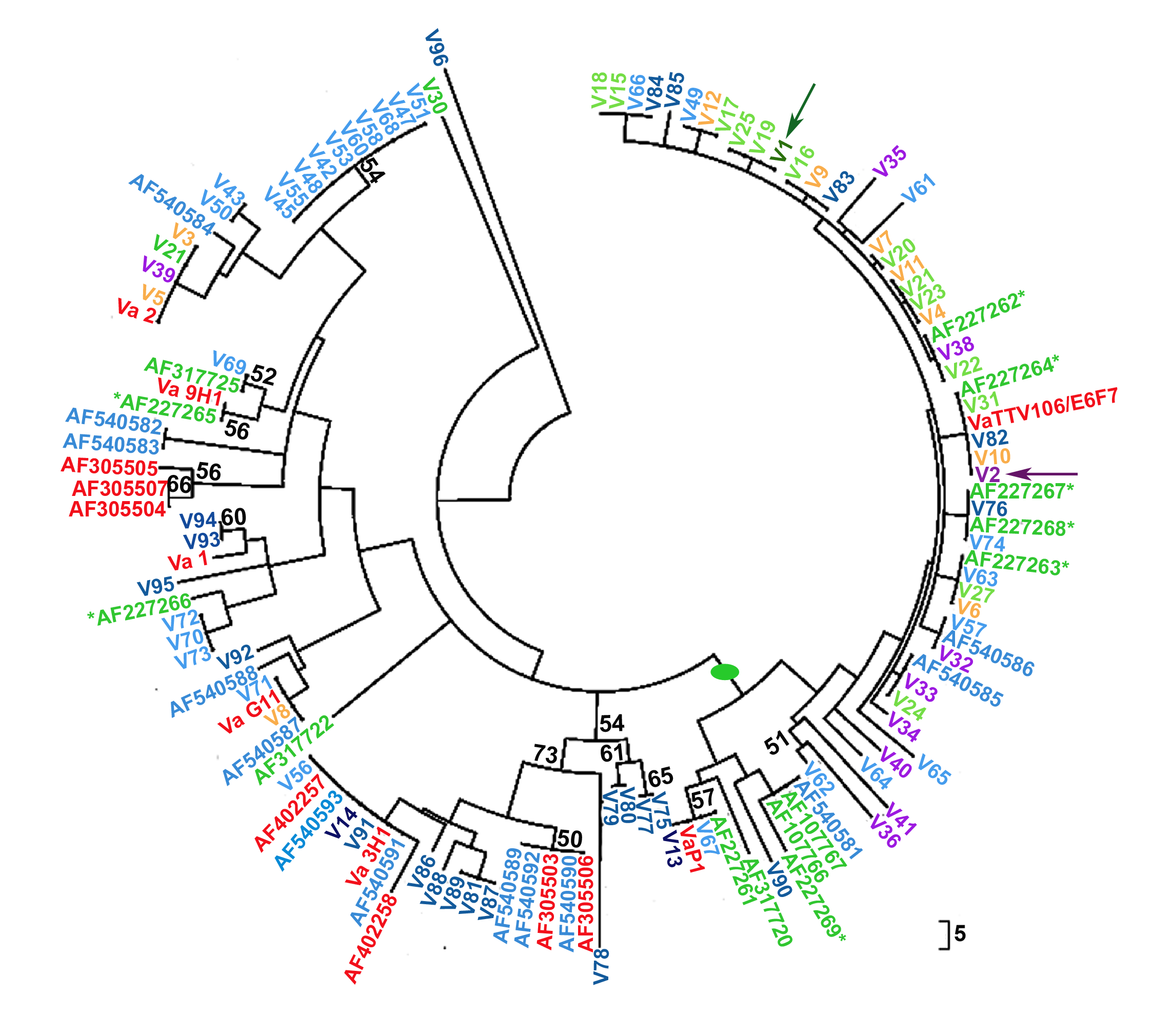

Supplement: Figure S1 — Phylogram of Msp 2 variants generated in vivo and in vitro. Neighbor joining tree generated using the amino acid sequences of the Msp2 HVR from several variants obtained during infection of Dermancentor spp. ticks, bovine hosts, and tick and mammalian cell cultures with several strains of A. marginale. HVRs are designated by their GenBank Accession numbers, along with the HVR from variants obtained in this study, designated by their variant code (e.g. V1, V2, etc). Variants generated during infection of salivary glands of ticks in vivo or during in vitro culture of A. marginale in tick cell lines are presented in light green. Predominant tick variant (V1) is shown in dark green and indicated by a green arrow. Variants marked with an asterisk (*) represent GenBank Msp2 sequences from A. marginale VA. Variants produced during acute phases of bovine infection in erythrocytes are shown in dark blue and those generated later during the persistent phase, or chronic infection, are shown in light blue. Mammalian cell culture variants are shown in purple. V2 is shown in dark purple and indicated by a purple arrow. Donor allele sequences are marked in red letters. Variants shared between the tick and mammalian cell cultures are shown in orange. Branch lengths show the number of amino acid changes between the variants. Values next to branches correspond to 3000 bootstrap analyses using MEGA 4.0. Only branches with over 50 percentage support are indicated in the tree. Vertical bar in lower right represents five amino acid changes. (TIF) [file pone.0036012.s001.tif]

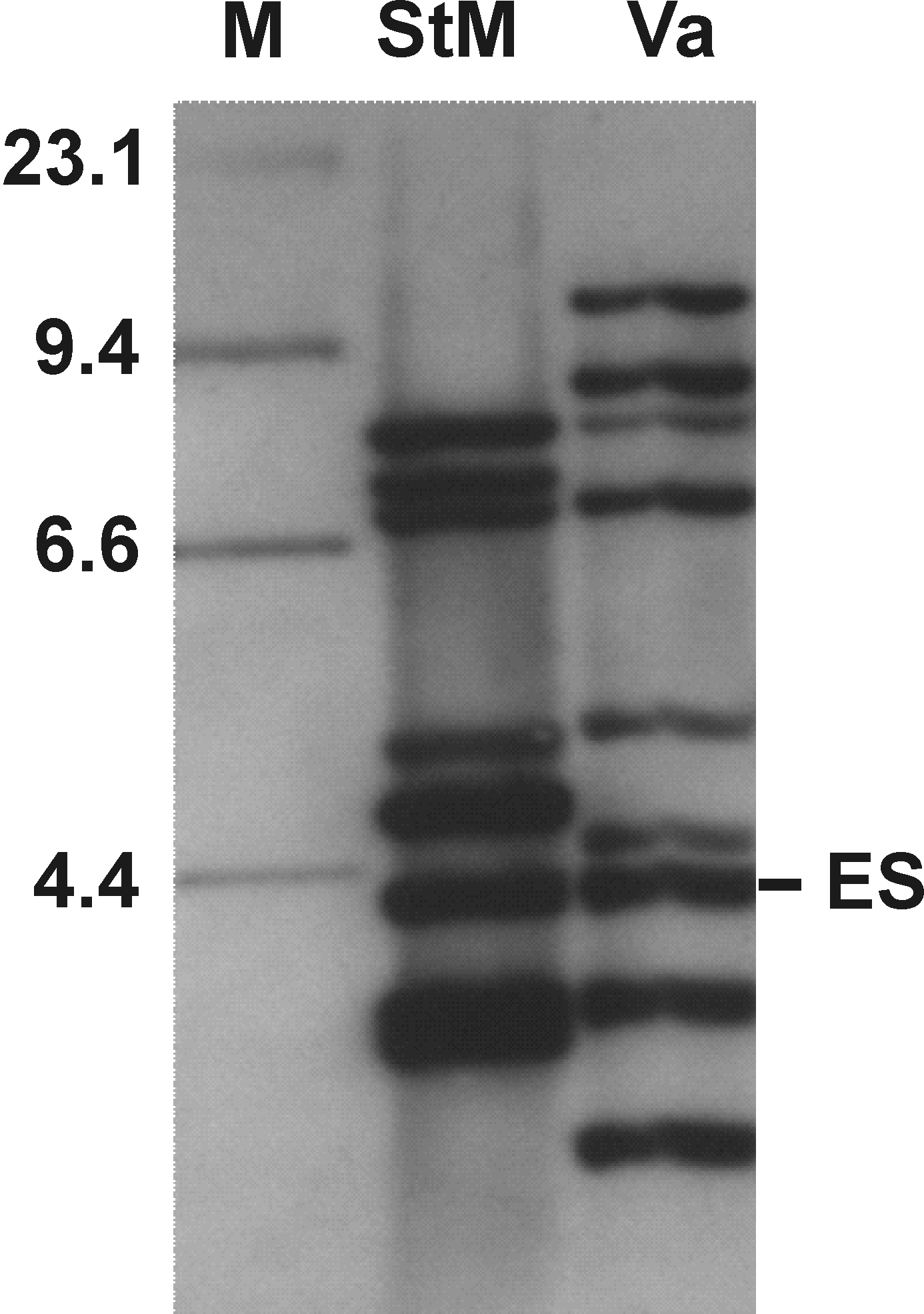

Supplement: Figure S2 — Genomic Southern analysis of msp2 . Lane M contains labeled lambda-HindIII markers with sizes (in kbp) indicated to the left, lanes StM and Va contain FspI digested genomic DNA from the St. Maries and A. marginale VA, respectively. The expression site is indicated (ES). (TIF) [file pone.0036012.s002.tif]

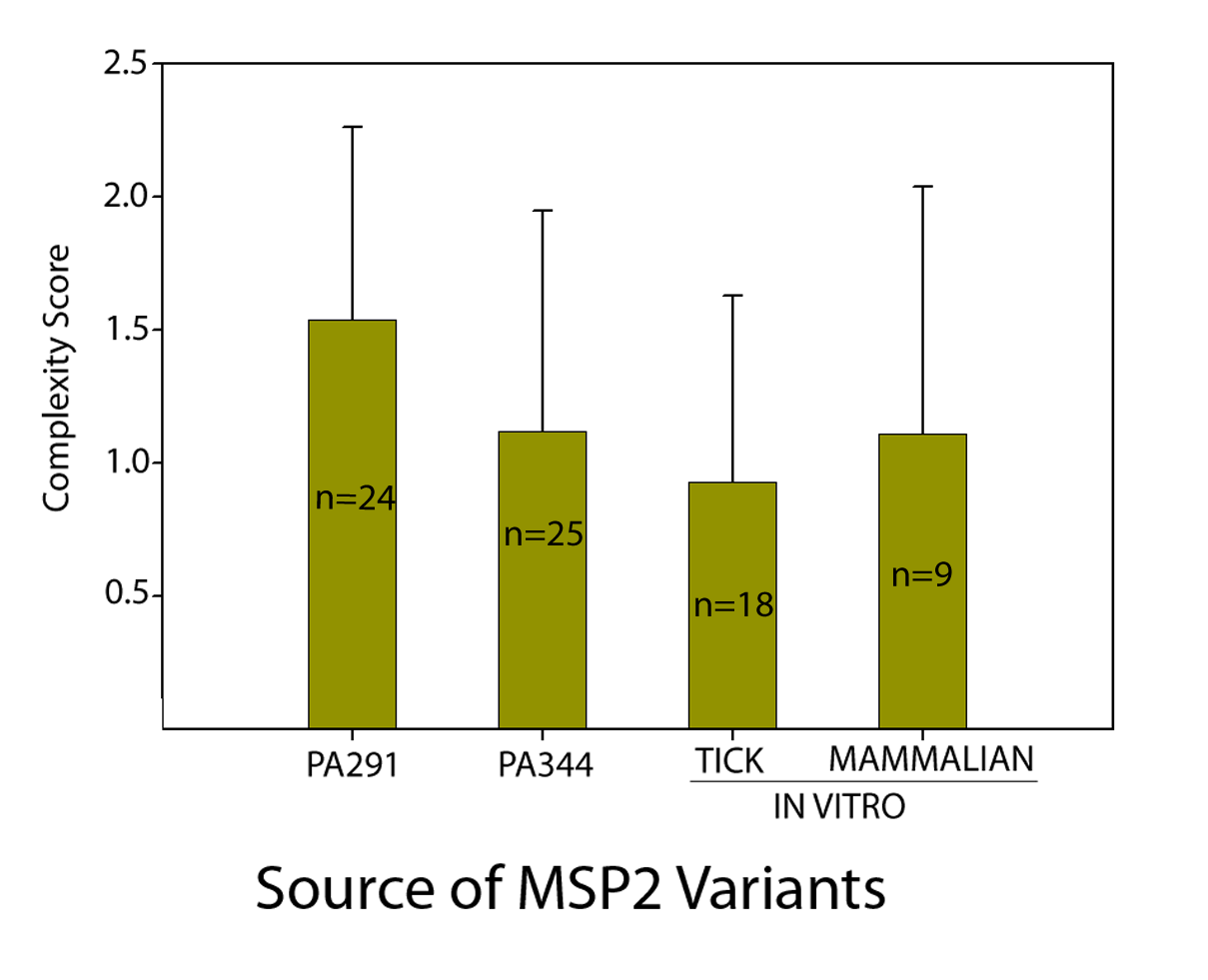

Supplement: Figure S3 — Changes in Msp2 complexity scores during infection of four different systems. Complexity was measured by determining the number of expression site segments derived from one of the eight different donor msp2 alleles encoded in the genome of A. marginale VA. Bars represent the average of the complexity score in a chronically infected animal (PA291), an acutely infected animal (PA344), and during culture in tick and mammalian cells. Lines represent the standard deviation of the mean. “n" stands for the number of variants present in each population that was used to calculate complexity scores. (TIF) [file pone.0036012.s003.tif]
