# Supplementary material for: Expression Patterns of Anaplasma marginale msp2 Variants Change in Response to Growth in Cattle, and Tick Cells versus Mammalian Cells
Source: PLoS One. 2012 Apr 25;7(4):e36012. doi: 10.1371/journal.pone.0036012 (PMC3338850; doi:10.1371/journal.pone.0036012)
Supplement: Materials and Methods S1 — (DOC) [file pone.0036012.s004.doc]

# Supplemental Materials and Methods S1

## *A. marginale Virginia strain growth in cultured cells*

For our studies on Msp2 expression, the *A. marginale* Virginia strain (*A. marginale* VA) (Kocan et al. 1980) was cultured in four tick cell lines and two mammalian cell lines. The tick cell lines used were BME26 (Esteves et al. 2008) derived from embryonated eggs of the southern cattle tick, *Rhipicephalus (Boophilus) microplus* (Canestrini), ISE6 (Munderloh et al. 1999) and IDE12 (Munderloh et al. 1994) derived from embryonated eggs of the blacklegged tick, *Ixodes scapularis* (Say), and DAE100T (Kurtti et al. 2005) from embryonated eggs of the Rocky Mountain wood tick, *Dermacentor andersoni* (Stiles). To confirm the species identity of the cell lines, the internal transcribed spacer 2 of the ribosomal operon was sequenced (GenBank accession numbers EU520392: EU520395) as described (Poucher et al. 1999). The mammalian cells used in this research were Vero (ATCC CCL-81 from kidney epithelial cells of an African Green monkey, *Cercopithecus aethiops*) and RF/6A cells (ATCC CRL-1780 from retina choroid endothelium of a rhesus monkey, *Macaca mulatta*). Cell cultures were maintained in L15B300 medium as described previously (Munderloh et al. 2004).

Uninfected tick cell cultures were inoculated with *A. marginale* VA (Munderloh et al. 1996) from ISE6 cells at passage 50. Mammalian cells were inoculated with *A. marginale* VA from ISE6 cells at passage 46. All cell lines were inoculated with cell free bacteria harvested from host cells disrupted by repeated passage through a 27 gauge needle fitted to a 5-ml syringe. Bacteria were purified by filtration through a 2.7 µm pore size filter, and added to cell cultures in five ml of medium. Subsequently, subcultures in each cell line were made when the percentage of *A. marginale* VA*-*infected cells reached >90% by passing one tenth of the volume of an infected cell culture onto a new cell layer.

***Bovine blood stabilates of A. marginale VA used for msp2 characterization***

Frozen blood stabilate from a splenectomised calf, PA291, infected with the *A. marginale* VA was regenerated from liquid nitrogen (Munderloh et al. 1996). This was the same stabilate used earlier to establish *A. marginale* VA in tick cell culture (Munderloh et al. 1996). The strain had been transferred from calf to calf 5 times by subinoculation of infected blood and was previously shown to be tick transmissible (Kocan et al. 1992). For comparison we used a second frozen blood stabilate taken from an infected 3 months old splenectomised Hereford calf, PA344, that had been inoculated with *A. marginale* VA grown in the *I. scapularis* cell line IDE8 cells. *A. marginale* VA had been passed two times and maintained in IDE8 cells for five months before being used to infect the calf (Munderloh et al. 1996). Blood was collected during rising parasitemia (2-3 weeks post inoculation). Frozen blood was rapidly thawed (37ºC ), transferred to a 15 ml tube containing 10 ml L15B medium and gently mixed. The cell suspension was centrifuged at 2000 x g for 10 min to remove DMSO and plasma. The cell pellet was resuspended in 10 ml L15B medium and the cells were washed twice more by centrifugation at 2000 x g for 10 min. DNA was extracted from the cell fraction as described below (*Cloning and analysis of msp2 variants expressed in vitro and in vivo*).

***Anaplasma culture and evaluation of differences in development***

After 5 - 6 passages of the bacteria in each of the cell lines, differences in the replication of *A. marginale* VA were assessed by measuring changes in the rate of cell-to-cell spread of the bacteria in each cell line (infection rate). Infection rate was calculated as the slope of the linear regression line fitted to the percent infected cells at different time points from day 3 until the culture reached >90% infection (3, 5, 7, and 12 days post-inoculation, and at subculture). Cultures were monitored by light microscopic observation of Giemsa-stained cells as described (Munderloh et al. 1996) and the percent infected cells at each time point was determined by counting a total of 300 cells in four replicates (each replicate representing a separate infected culture) per cell line. Statistical differences in growth rates were evaluated using Repeated Measures ANOVA with SigmaPlot (Systat Software, Inc., San José, California).

***Western blots***

Expression of Msp2 variants during infection of the different cell lines was analyzed by Western blotting. Protein was extracted from 2.7µm pore-size filter-purified bacteria from each cell line suspended in sample buffer (0.125 M Tris buffer-2% SDS, 660 mM β-mercaptoethanol and 10% bromophenol blue) and boiled for 5 min. Negative controls consisted of proteins extracted from uninfected IDE12 and ISE6 cells. Protein concentrations were measured using the RC DC^TM^ protein assay (Bio-Rad, Hercules, California) in a BioPhotometer (Eppendorf, Hamburg, Germany). Protein samples (30 ng per well) and 7 µl of SeeBlue® plus 2 protein size markers (Invitrogen, Carlsbad, California) were resolved in SDS-polyacrylamide, 8 to 16% gradient gels (ISC BioExpress, Kaysville, Utah) by electrophoresis in Tris-glycine buffer for 1.5 hours at 100 V. Gels were blotted onto Immobilon-P membranes (Millipore, Bedford, Massachusetts) at 70 V for 2 hours, and blocked with 5% nonfat dry milk in PBS at 6ºC overnight. Blots were incubated with various Msp2 specific antibodies (Table 1) diluted in PBS with 3% bovine serum albumin overnight at 6ºC, washed four times in PBS, and labelled with anti-rabbit or anti-mouse IgG conjugated to horseradish peroxidase, as required. Blots were developed with the ImmunoPure® metal enhanced DAB substrate (Pierce, Rockford, Illinois) system.

## *Immuno-fluorescence assay~~s~~ (IFA)*

Samples from cell cultures infected with *A. marginale* VA at 50% were spun onto microscope slides for 5 min at 60 x g, using a Cytospin (Shandon Southern Instruments, Sewickley, Pennsylvania). This was repeated with three different culture flasks for each cell line. The slides were air-dried, fixed twice in methanol for five min, and dried for 30 min at 50 ºC. The cell spots were then incubated with primary antibody (Table 1) for one hr at room temperature to determine the proportion of *A. marginale* VA colonies expressing Msp2 variants,,and labelled with FITC-conjugated secondary antibody of the appropriate species specificity for another hr at room temperature. Uninfected cells and infected cells not exposed to primary antibody served as controls. The slides were dried, the cell spots were counter-stained with Evan’s blue (0.0005%) and covered with VectaShield mounting medium with 4'-6-Diamidino-2-phenylindole (DAPI) (Vector laboratories, Burlingame, California). DAPI was used to stain DNA from all *A. marginale* VA and host cell nuclei. The percentage of colonies that bound a specific antibody was determined by counting 100 infected cells (counted using the host nuclei). Two-way ANOVA (SigmaPlot; Systat Software, Inc., San José, California) was carried out to evaluate the significance of the differences in antibody binding between *A. marginale* VA growing in the various cell lines.

***Cloning and analysis of msp2 variants expressed in vitro and in vivo***

After approximately 20 continuous passages in the same cell line, *A. marginale* VA were harvested from host cells and filter-purified as described above, and collected at 16,000 x g for 1 min at 4 ºC. Bacterial DNA was extracted according to the tissue culture protocol of the Gentra Puregene genomic DNA kit (Qiagen, Valencia, California) from each of one infected tick and mammalian cell culture grown in 25-cm^2^ flasks. To confirm infection, *A. marginale* VA was PCR-amplified using primers specific for the *16s rRNA* gene of *Anaplasma* and *Ehrlichia spp.* and primers specific to the *msp1α* gene of *A. marginale* (Table 2).

We additionally extracted DNA from two bovine blood samples. PA291 blood sample was analyzed to determine whether the variants seen during culture in tick or mammalian cells were already present in the original inocula. This was done to determine if the culture variants were completely independent from the original set of variants and arose in the absence of immune selection. PA291 DNA was additionally treated with GeneReleaser, according to manufacturer’s specifications (BioVentures Inc., Murfreesboro, Tennessee). The second DNA sample from animal PA344 was extracted using the whole blood protocol Gentra DNA kit previously described. This blood sample was sequenced to determine whether or not variants from the tick cell culture were present during the acute phase of infection or if the cultured repertoire of variants were generated completely *de* *novo*.

The *msp2* gene copy in the expression locus from the *A. marginale* VA populations growing in each cell line was amplified using the primers MSP2 Forward and MSP2 Reverse (Table 2), following the PCR conditions as described (Futse et al. 2005). Reaction mixtures of 50 µl contained 1X Pfu buffer, 0.2 mM of each dNTP, 1 µM of each primer, 2.5 units of Pfu DNA polymerase (Promega, Madison, Wisconsin), and 100 ng of template DNA*.* Amplified products were cloned into pCR4®-TOPO (Invitrogen, Grand Island, New York). For comparison, DNA from *A. marginale* VA passage 46 in ISE6 cells (the passage used as starting inoculum for cultivation in mammalian cells) was extracted and *msp2* sequences in the expression locus amplified and cloned the same way. Thirty plasmid inserts were analyzed to identify the predominant variant present prior to cultivation in the various cell lines. Analysis of 30 clones provided >90% probability of detecting variants present in the population at greater than 5% (Palmer et al. 2007).

To analyze the variation in *msp2* expression at the molecular level, DNA from 120 clones from each of the tick and mammalian cell lines and the two blood samples was isolated and sequenced using the BioMedical Genomics Center facilities (University of Minnesota). This number of clones was sufficient to obtain even rare variants that are present in small percentages in each cell line. The predominant variant was determined by counting the number of plasmids that contained this variant, and was calculated as a percentage of predominance by dividing this number by the total number of plasmids analyzed (in this case 120).

Nucleotide sequences were analyzed and translated into amino acid sequences using MacVector 10.6 (MacVector Inc., Cary, North Carolina) and Sequencher 4.8 (Gene Codes Corporation, Ann Arbor, Michigan). The HVR of the amino acid sequences were aligned and compared using ClustalW. Additional, *msp2* sequences cloned by several investigators from ticks, tick cells, and blood samples from acute and persistently infected animals infected with *A. marginale* VA, South Idaho, or Oklahoma strains (Rurangirwa et al. 1999; Rurangirwa et al. 2000; Barbet et al. 2001) were compared with the sequences recovered in this study. The sequences of the *msp2* donor alleles of *A. marginale* VA and South Idaho strains (Brayton et al. 2001) were included in the phylogenetic analysis of all the variants. Translated variants were subject to phylogenetic analysis using the total number of differences in MEGA 4.0 (Tamura et al. 2007) and Neighbor-Joining with 3000 bootstrap replicates to examine the relationship of variants to each other.

***Determination of msp2 donor allele repertoires:***

Genomic DNA was extracted from *A. marginale* VA strain infected blood using the Gentra Puregene DNA isolation kit (Qiagen). As previous studies have indicated that the *A. marginale* *msp2* allelic repertoires are positionally conserved between strains (Dark et al. 2009), we employed a locus specific PCR strategy using eight locus specific primer sets designated: P1, ψ1, ψ2, G11, 3H1, E6F7, 9H1, and TTV106 (Table 3). The PCR Master kit (Roche, Indianapolis, Indiana) was used as follows: 30 cycles of melting at 94oC for 30 s, variable annealing temperature for 30 s, and extension at 72°C (annealing temperatures and extension times are shown in Table 3). The PCR amplicons were ligated into the pCR4- TOPO vector using the TOPO-TA cloning kit (Invitrogen, Grand Island, New York), and then transformed into TOP10 *E. coli* cells. DNA was extracted by using the Wizard plus SV Miniprep DNA purification system (Promega, Madison, Wisconsin). The plasmid DNA was sequenced with the Big Dye kit (Applied Biosystems, Carlsbad, California). Inserts for the P1, G11, 9H1, and TTV106 loci were sequenced using HV univ for and HV univ rev primers (Rodriguez et al. 2005), and 3H1, E6F7, ψ1, and ψ2 were sequenced using T3 and T7 primers. The *A. marginale* VA donor alleles have been deposited in GenBank with accession numbers VaP1: JN703159, VaG11: JN703155, Va9H1: JN703157, Va3H1: JN703158, VaE6F7: JN703156, Vaψ1: JN703161, and Vaψ2: JN703160 and VaTTV106: JN703154.

***Bioinformatic and complexity analysis of Msp2 variants***

Several parameters including charge, amino acid composition, and grand average of hydropathicity (GRAVY) were analyzed using ProtParam from the ExPASy proteomics server (www.expasy.ch/tools/protparam.html). The secondary structure of the variants was predicted using Psipred (<http://bioinf.cs.ucl.ac.uk/psipred/>) and the Chou-Fasman method from MacVector®. The differences in structure between variants and their frequency was analysed to determine if patterns in variation coincided with changes in the potential secondary structure.

Additionally, complexity scores and donor allele usage were determined as previously described (Futse et al. 2005; Futse et al. 2009). Translated amino acid sequences of variants obtained from *in vitro* tick and mammalian cultures as well as of variants in PA291 and PA344 were aligned and compared with each of the eight donor alleles of *A. marginale* VA. Preferential donor allele usage was evaluated by determining the frequency of recombination of whole or segments of specific donor alleles during production of new variants as described (Futse et al. 2009). This is an indicator of the frequency at which a donor allele is used for the production of variants, whether or not this donor allele provides a fitness advantage to the organisms. We used the chi-square test to validate non-random use of donor alleles in the generation of expressed *msp2* variants.

To calculate differences in donor allele usage within a population were considered the predominance of all variants containing a particular donor allele sequence. The usage of one donor allele in the population was determined as the sum of the predominance of all the variants containing complete or partial sequences of that particular donor allele’s HVR. The number of *E. coli* clones containing a particular variant was considered to represent the frequency of use of the variant in the whole population (described in the section *Cloning and analysis of msp2 variants expressed in vitro and in vivo*). Significance was evaluated using chi-square.

***Analysis of differences in msp2 expression by qRT-PCR***

RNA from *A. marginale* grown in ISE6, DAE100T, and RF/6A cells was extracted using the Absolutely RNA miniprep kit (Stratagene, La Jolla, California) and treated with Turbo DNA-free DNase (Ambion, Austin, Texas) to eliminate any remaining DNA. qRT-PCR was performed using primers Msp2f and Msp2r (Table 2), that are complementary to the conserved flanking regions of *msp2* at positions 4798 - 4817 bp and 5163 – 5144, respectively, of the *A. marginale* VA *msp2* operon GenBank accession number: AY132312). *msp2* expression was normlized against expression of RNA polymerase B *(rpoB)* and 16s rRNA genes. All reactions were performed in a Stratagene Mx3005 QPCR machine using the Brilliant II SYBR green QRT-PCR Master Mix 1-Step kit (Stratagene, La Jolla, California) with a primer concentration of 200 nM, 30 nM ROX (as reference dye), and 100 ng of total RNA. Cycling conditions were as follow: an initial cycle of reverse transcription for 30 min at 50 ºC, follow by a denaturalization cycle of 10 min at 95 ºC, and 40 cycles with denaturalization at 95 ºC for 30 seconds, an annealing temperature of 63 ºC for 1 min, and extension at 72 ºC for 1 min, with a final denaturalization at 95 ºC and annealing for 30 seconds at 63 ºC to determine the dissociation curve. The comparative Ct method was used to calculate the fold change in expression between the different cell lines. Statistical significance of gene expression results was evaluated using REST© 2008 (Pfaffl et al. 2002) (<http://www.gene-quantification.de/rest-2008.html>), with 2000 randomizations.

## *Accession numbers for Msp2 variants*

The predominant *msp2* variant from tick cell lines was designated as V1 (EU496530), whereas the predominant variant from mammalian cells was designated as V2 (EU496531), the *msp2* variant shared in tick cell lines only was designated V4 (EU496533), and variants shared between the mammalian and tick systems were designated V3 (EU496532) and V5 – V12 (JQ082310, EU512243, EU496534, EU517681, EU499673, JQ177150, EU499670). Variants shared between blood samples were designated V13 (EU627153) and V14 (EU627154). *msp2* variants unique to the tick cell line ISE6 were assigned codes V15 to V17 (EU496539, JQ082308, JQ082309). Variants unique to BME26, DAE100T and IDE12 cells were coded V18 to V21 (EU496538, EU496536, JQ082306, JQ082307), V22 to V26 (JQ177151 – JQ177155), and V27 to V31 (EU499671, EU499672, JQ177156, JQ177157) respectively. Variants unique to the mammalian cell lines Vero and RF/6A cells were coded V32 to V36 (EU512244 – EU512248), and V38 to V41 (EU517678, EU517679, EU512237, EU512238), respectively. *msp2* variants from PA344 were assigned codes V42 to V74 (EU517683-EU517694, EU526866-EU526882), and variants from PA291 were given the codes V75 to V97 (EU526883– EU526897, EU627148- EU627155).

# References

Barbet AF, Yi J, Lundgren A, McEwen BR, Blouin EF et al. (2001) Antigenic variation of *Anaplasma marginale*: major surface protein 2 diversity during cyclic transmission between ticks and cattle. Infect Immun 69(5): 3057-3066.

Brayton KA, Knowles DP, McGuire TC, Palmer GH (2001) Efficient use of a small genome to generate antigenic diversity in tick-borne ehrlichial pathogens. Proc Natl Acad Sci U S A 98(7): 4130-4135.

Dark MJ, Herndon DR, Kappmeyer LS, Gonzales MP, Nordeen E et al. (2009) Conservation in the face of diversity: multistrain analysis of an intracellular bacterium. BMC Genomics 10(16): 16.

Esteves E, Lara FA, Lorenzini DM, Costa GH, Fukuzawa AH et al. (2008) Cellular and molecular characterization of an embryonic cell line (BME26) from the tick *Rhipicephalus (Boophilus) microplus*. Insect Biochem Mol Biol 38(5): 568-580.

Futse JE, Brayton KA, Knowles DP, Jr., Palmer GH (2005) Structural basis for segmental gene conversion in generation of *Anaplasma marginale* outer membrane protein variants. Mol Microbiol 57(1): 212-221.

Futse JE, Brayton KA, Nydam SD, Palmer GH (2009) Generation of antigenic variants via gene conversion: Evidence for recombination fitness selection at the locus level in *Anaplasma marginale*. Infect Immun 77(8): 3181-3187.

Kocan KM, Teel KD, Hair JA (1980) Demonstration of *Anaplasma marginale* Theiler in ticks by tick transmission, animal inoculation, and fluorescent antibody studies. Am J Vet Res 41(2): 183-186.

Kocan KM, Stiller D, Goff WL, Claypool PL, Edwards W et al. (1992) Development of *Anaplasma marginale* in male *Dermacentor andersoni* transferred from parasitemic to susceptible cattle. Am J Vet Res 53(4): 499-507.

Kurtti TJ, Simser JA, Baldridge GD, Palmer AT, Munderloh UG (2005) Factors influencing in vitro infectivity and growth of *Rickettsia peacockii* (Rickettsiales: Rickettsiaceae), an endosymbiont of the Rocky Mountain wood tick, *Dermacentor andersoni* (Acari, Ixodidae). J Invertebr Pathol 90(3): 177-186.

Munderloh UG, Liu Y, Wang M, Chen C, Kurtti TJ (1994) Establishment, maintenance and description of cell lines from the tick *Ixodes scapularis*. J Parasitol 80(4): 533-543.

Munderloh UG, Blouin EF, Kocan KM, Ge NL, Edwards WL et al. (1996) Establishment of the tick (Acari:Ixodidae)-borne cattle pathogen *Anaplasma marginale* (Rickettsiales:Anaplasmataceae) in tick cell culture. J Med Entomol 33(4): 656-664.

Munderloh UG, Lynch MJ, Herron MJ, Palmer AT, Kurtti TJ et al. (2004) Infection of endothelial cells with *Anaplasma marginale* and *A. phagocytophilum*. Vet Microbiol 101(1): 53-64.

Munderloh UG, Jauron SD, Fingerle V, Leitritz L, Hayes SF et al. (1999) Invasion and intracellular development of the human granulocytic ehrlichiosis agent in tick cell culture. J Clin Microbiol 37(8): 2518-2524.

Palmer GH, Futse JE, Leverich CK, Knowles DP, Jr., Rurangirwa FR et al. (2007) Selection for simple major surface protein 2 variants during *Anaplasma marginale* transmission to immunologically naive animals. Infect Immun 75(3): 1502-1506.

Pfaffl MW, Horgan GW, Dempfle L (2002) Relative expression software tool (REST) for group-wise comparison and statistical analysis of relative expression results in real-time PCR. Nucleic Acids Res 30(9): e36.

Poucher KL, Hutcheson HJ, Keirans JE, Durden LA, Black WCt (1999) Molecular genetic key for the identification of 17 Ixodes species of the United States (Acari:Ixodidae): a methods model. J Parasitol 85(4): 623-629.

Rodriguez JL, Palmer GH, Knowles DP, Jr., Brayton KA (2005) Distinctly different msp2 pseudogene repertoires in *Anaplasma marginale* strains that are capable of superinfection. Gene 361: 127-132.

Rurangirwa FR, Stiller D, Palmer GH (2000) Strain diversity in major surface protein 2 expression during tick transmission of *Anaplasma marginale*. Infect Immun 68(5): 3023-3027.

Rurangirwa FR, Stiller D, French DM, Palmer GH (1999) Restriction of major surface protein 2 (MSP2) variants during tick transmission of the ehrlichia *Anaplasma marginale*. Proc Natl Acad Sci U S A 96(6): 3171-3176.

Tamura K, Dudley J, Nei M, Kumar S (2007) MEGA4: Molecular Evolutionary Genetics Analysis (MEGA) software version 4.0. Mol Biol Evol 24(8): 1596-1599.
