# Supplementary material for: Expression Patterns of Anaplasma marginale msp2 Variants Change in Response to Growth in Cattle, and Tick Cells versus Mammalian Cells
Source: PLoS One. 2012 Apr 25;7(4):e36012. doi: 10.1371/journal.pone.0036012 (PMC3338850; doi:10.1371/journal.pone.0036012)
Supplement: File S1 — Abstract in Spanish. (DOC) [file pone.0036012.s005.doc]

# Sumario

Variación antigénica de proteínas abundantes en la superficie de patógenos es considerada una maniobra que ellos usan para evadir la respuesta inmune del hospedero, y está presente en patógenos tan diferentes como lo son bacterias y protozoos. La proteína “major surface protein 2” (Msp2) del patógeno *Anaplasma marginale*, transmitido por garrapatas, está envuelta en variación antigénica. Este proceso dinámico también ocurre durante la infección de su vector, garrapatas, y en la ausencia de presión inmune. Examinamos variantes de Msp2 expresadas durante la infección de cuatro líneas de garrapatas y dos líneas de células mamíferas para determinar si las variantes expresadas están correlacionadas con la célula hospedera. Las colonias de *Anaplasma marginale* se desarrollaron de una manera diferente en cada una de las líneas de células (P<0.001). Utilizando dos anticuerpos mono-específicos y uno monoclonal en contra de Msp2, detectamos la expresión de variantes con diferencias en su peso molecular de acuerdo a Western-blots. Pruebas inmunofluorescentes revelaron que específicos anticuerpos reconocieron 25 a 60% de las colonias, dependiendo de la célula hospedera (P<0.001). Análisis molecular de las variantes expresadas durante la infección de las células demostró la dominancia de una variante (V1) durante infección de células de garrapatas y una diferente (V2) en células de mamíferos. Análisis de la estructura putativa de las variantes reveló un cambio en la estructura de la proteína cuando *A. marginale* fue transferida de un tipo de célula a la otra, sugiriendo que la expresión de ciertas variantes de Msp2 depende del tipo de la célula (garrapata o mamífera) en la cual se desarrolla. El mismo análisis de la estructura putativa de más de 200 variantes de Msp2 en garrapatas, muestras de sangre y otras células, disponibles en GenBank, demostró la dominancia de una estructura específica durante la infección de un hospedero. Esto sugirió que selección de ciertas estructuras posiblemente también ocurre *in vivo*. La selección de una estructura en proteínas de la superficie de este patógeno puede indicar que Msp2 llena un rol importante durante la infección y adaptación a sistemas diferentes.
